# Computer vision and machine learning enabled soybean root phenotyping pipeline

**DOI:** 10.1186/s13007-019-0550-5

**Published:** 2020-01-23

**Authors:** Kevin G. Falk, Talukder Z. Jubery, Seyed V. Mirnezami, Kyle A. Parmley, Soumik Sarkar, Arti Singh, Baskar Ganapathysubramanian, Asheesh K. Singh

**Affiliations:** 1 Department of Agronomy, Iowa State University, Ames, USA; 2 Department of Mechanical Engineering, Iowa State University, Ames, USA

**Keywords:** RSA, Root, Phenotyping, Phenomics, Computer vision, Machine learning, Breeding, Soybean, Time series, Image analysis

## Abstract

**Background:**

Root system architecture (RSA) traits are of interest for breeding selection; however, measurement of these traits is difficult, resource intensive, and results in large variability. The advent of computer vision and machine learning (ML) enabled trait extraction and measurement has renewed interest in utilizing RSA traits for genetic enhancement to develop more robust and resilient crop cultivars. We developed a mobile, low-cost, and high-resolution root phenotyping system composed of an imaging platform with computer vision and ML based segmentation approach to establish a seamless end-to-end pipeline - from obtaining large quantities of root samples through image based trait processing and analysis.

**Results:**

This high throughput phenotyping system, which has the capacity to handle hundreds to thousands of plants, integrates time series image capture coupled with automated image processing that uses optical character recognition (OCR) to identify seedlings via barcode, followed by robust segmentation integrating convolutional auto-encoder (CAE) method prior to feature extraction. The pipeline includes an updated and customized version of the Automatic Root Imaging Analysis (ARIA) root phenotyping software. Using this system, we studied diverse soybean accessions from a wide geographical distribution and report genetic variability for RSA traits, including root shape, length, number, mass, and angle.

**Conclusions:**

This system provides a high-throughput, cost effective, non-destructive methodology that delivers biologically relevant time-series data on root growth and development for phenomics, genomics, and plant breeding applications. This phenotyping platform is designed to quantify root traits and rank genotypes in a common environment thereby serving as a selection tool for use in plant breeding. Root phenotyping platforms and image based phenotyping are essential to mirror the current focus on shoot phenotyping in breeding efforts.

## Background

Over the past century, classical and technology-driven breeding techniques have aimed to achieve higher seed yield in major crops. The increase in seed yield comes concomitantly with a focus on improving the agronomic, disease protection and other perceptible traits that are expressed and observable above ground. Root system architecture (RSA), or the spatial arrangement of the root and its components [1], functions to provide water and nutrient acquisition, nutrient storage, anchorage and to foster plant–microbe interactions such as nodulation in N-fixing crops, which are relatively inconspicuous yet fundamental to plants’ performance and are indirectly selected traits in breeding programs particularly for non-tuber or root crops [2]. Root structure also correlates to environmental advantages, such as nutrient acquisition [1, 3], drought [4–7], flood tolerance [8], and lodging resistance [9].

Plant breeders have continually modified the above-ground features of the plant as these have been the easier to select; however, the hidden-half of the plant warrants further investigation for major agronomic crops including soybean (*Glycine max *L.* Merr.*), maize (*Zea mays *L.), wheat (*Triticum aestivum *L.) and rice (*Oryza sativa *L.) [5, 10]. This limitation in selecting for root phenotypes in soybean and other pulse, oilseed and cereal crop species arises from the difficulty of root trait measurement, and therefore the inability to study and utilize root architecture, morphology, topology, distribution within the soil, response to environmental stimuli and growth over time [1, 11–14]

Root structure correlates to environmental advantages, such as nutrient acquisition [1, 3], drought [4–7], flood tolerance [8], and lodging resistance [9]. Root phenotyping and further research is hindered due to wide technological gap in our ability to collect, observe and quantify important root trait data which is exacerbated by trait genetic complexity [15–18], phenotypic expression complexity [19], morphometric nature of their expression [20], and environmental interaction including soil structure [20], nutrient availability [3], temperature [21], water [22], interactions with other plants [2, 23] and microbes [24].

Researchers have generally taken one of three strategies to approach root phenotyping: including (1) controlled laboratory methods [18, 25, 26], (2) moderately controlled greenhouse methods [27, 28] and (3) minimally controlled field methods [29–32]. While the complexity of environment becomes more relevant to field scale production and physiological relevance with field methods, controlled laboratory methods are amenable to large scale phenotyping and throughput; therefore, researchers continue to explore ways to bridge the gap of lab versus field methods [11]. The existing major impediment is the high labor and time costs in the field for root trait phenotyping [29, 32]. This motivates our research to enable automation and increase throughput of root trait studies. The ability to study larger sample sizes will provide exciting opportunities to understand the role of RSA and its and application in future research.

### Breeding for root system architecture (RSA) traits

Root system architecture is a complex of polygenic traits consisting of sub-root system parameterizations such as root growth habit, total root length, primary root length, root number, root angle, root thickness, root length density (root extension and distribution), root surface area, and are paramount in improving plant performance and seed yield [2, 23]. Monocot and dicots have distinct morphological parameters that are used to classify their roots into fibrous and taproot growth types, respectively. Due to the difficulty associated with the measurement of RSA traits and the high level of morphological plasticity of roots in soil [33–36], breeding programs rarely utilize RSA traits as a method of selection [5]. Furthermore, RSA traits remain elusive in plant breeding selection practices due to the RSA plasticity caused by environmental variation, lack of cost effective field plot root extraction protocols, and limited appropriate phenotyping platforms and tools [5, 37]. Identification of genes which control QTL (quantitative trait loci) for RSA has come with minimal success demonstrating that further genetics research is needed [38, 39].

Researchers have noted diversity of RSA within crop species such as maize [40], soybean [5, 41–44], common bean (*Phaseolus vulgaris *L.) [45], rice [26, 46] and wheat [47]. It is important to note that the North American soybean genepool is very narrow [48], therefore the expected gain from RSA traits could be high and rewarding, but will rely on the identification and incorporation of genetic diversity [49–51].

Before plant breeders can select for RSA traits, available genetic and phenotypic diversity needs to be explored and characterized. Therefore, accurate and efficient quantification of root architecture traits and diversity as well as associated physiological processes, is a pertinent requirement for addressing breeding objectives. Fortuitously, in the current era of phenomics and big data there is a continual advancement in high-throughput phenotyping (HTP) methods that can enhance researcher’s ability to assess above and below ground organs and traits. New technological innovation in computers, sensors, robotics and data analytics, including computer vision [52, 53], automation [54], remote sensing [55], ML [56] and deep learning (DL) [57] have allowed breeders and researchers to capture high resolution and high dimensional attributes of diverse phenotypic data non-destructively on a vast spatio-temporal scale [56, 58, 59]. These include primarily above ground traits [60–65] and to a lesser extent, root related traits [66–68]. However, continual efforts are needed to decipher the genetics of root traits to realize the genetic potential of root trait driven breeding. With phenomic information on both root and shoot traits, plant scientists will be empowered to deploy above and below ground phenotypes optimized to targeted climactic conditions and agronomic management techniques.

Technological challenges in RSA trait phenotyping can be divided into two major components: (1) root extraction from soil (for review, see [69]), and (2) imaging and computer aided feature (trait) extraction [70]. This dictates a need for advances in imaging protocols, computer vision and ML for trait extraction. Conventional approaches for root examination include field extractions [32], soil coring [13, 71] and minirhizotrons [72], but advances in X-ray computed tomography [68, 73, 74], magnetic resonance imaging (MRI) and positron emission tomography (PET) [21, 75], and 3D imaging approaches [26, 76, 77] have helped obtain higher resolution root trait data. The low throughput and high cost often prevent integration of these approaches in large scale genetic material screening [11, 12, 59, 69, 78]. At the onset, a reduction of cost and time are imperative to scaling plant phenotyping methodologies and require standardized protocols. There is little standardization on physical platforms (hardware) used for image based root phenotyping. However, for image analytics, several software tools are currently available that extract data through analyses of high resolution digital images with advanced computer analysis. This non-exhaustive list includes: archiDART [79], ARIA [80], DART [81], DIRT [82], EZ-Rhizo [83], GiA Roots [84], GLO-Roots [85], RhizoChamber-Monitor [86], RootNav [87], RootReader2D, RootSystemAnalyzer [88], RooTrak [68], RootTrace [89], SmartRoot [66]. These freeware such as ARIA (Automatic Root Image Analysis), have been developed to be faster and more adaptable to the alternative industry standard software WinRHIZO [80]. Advances in computer vision and image analytics have made feature extraction efficient, effective, accurate, and potentially non-destructive. The recent software are also multi-functional due to their ability to perform fast processing based on digital images, generation of information on various traits, with higher throughput [80].

Recent coupling of computer vision with ML has facilitated the generation of software tools that include automated learning for image preprocessing, image processing and feature extraction that will aid to reduce measurement variability and remove subjectivity and biases. Alone, computer vision enables software to identify objects and structures within images; while ML has been deployed to learn and classify those objects or structures [90]. In recent root architecture studies, researchers trained their model to recognize and differentiate root tips from 2D images in an automated process [91]. Other studies used a random forest based approach to replace missing trait values in highly noisy root images [92]. Nevertheless, with the strides being made in software, data processing, and phenotyping protocols, a methodology is needed that is low-cost, scalable, and robust to diverse phenotypes and experimentation to begin standardizing RSA trait acquisition.

In this paper, we describe hardware, software and analytical solutions for an end-to-end controlled environment soybean root phenotyping pipeline. The main objectives were to develop: (1) a low barrier to entry system facilitating the growth and imaging of hundreds of plants, (2) a computer vision program to automate image capture and curation, (3) image segmentation using heuristic and ML approaches and (4) a software tool to automate the extraction of a multitude of seedling RSA traits. The final product is an end-to-end pipeline with a fully automated software complete with tunable image thresholding and image based trait extraction. To summarize, the pipeline provides non-destructive evaluation of a large number of soybean genotypes in controlled conditions in a rapid manner at lowered cost of phenotyping alleviating the phenotyping bottleneck thus enhancing research and breeding progress related to RSA. We envision that this combination of phenotyping platform and data analytics will meet the needs of various users regardless of technical experience.

## Methods

### Plant material

For this study, 292 genotypes comprising a subset of the USDA soybean core collection and a subset of Soybean Nested Association Mapping (SoyNAM) parents were selected and were previously genotyped [93, 94]. For the purpose of this manuscript, we restrict our analyses and results presentation to 115 maturity group II (MG 2) genotypes to target the local Iowa environment for within maturity group comparisons. These genotypes consisted of a wide range in geographical origin (12 countries) and growth habit (determinate, semi-determinate, indeterminate) along with various other morphological and seed quality traits to meet our requirement for a diverse set of lines to test during hardware and software development.

### Growing protocol

**Fig. 1:**
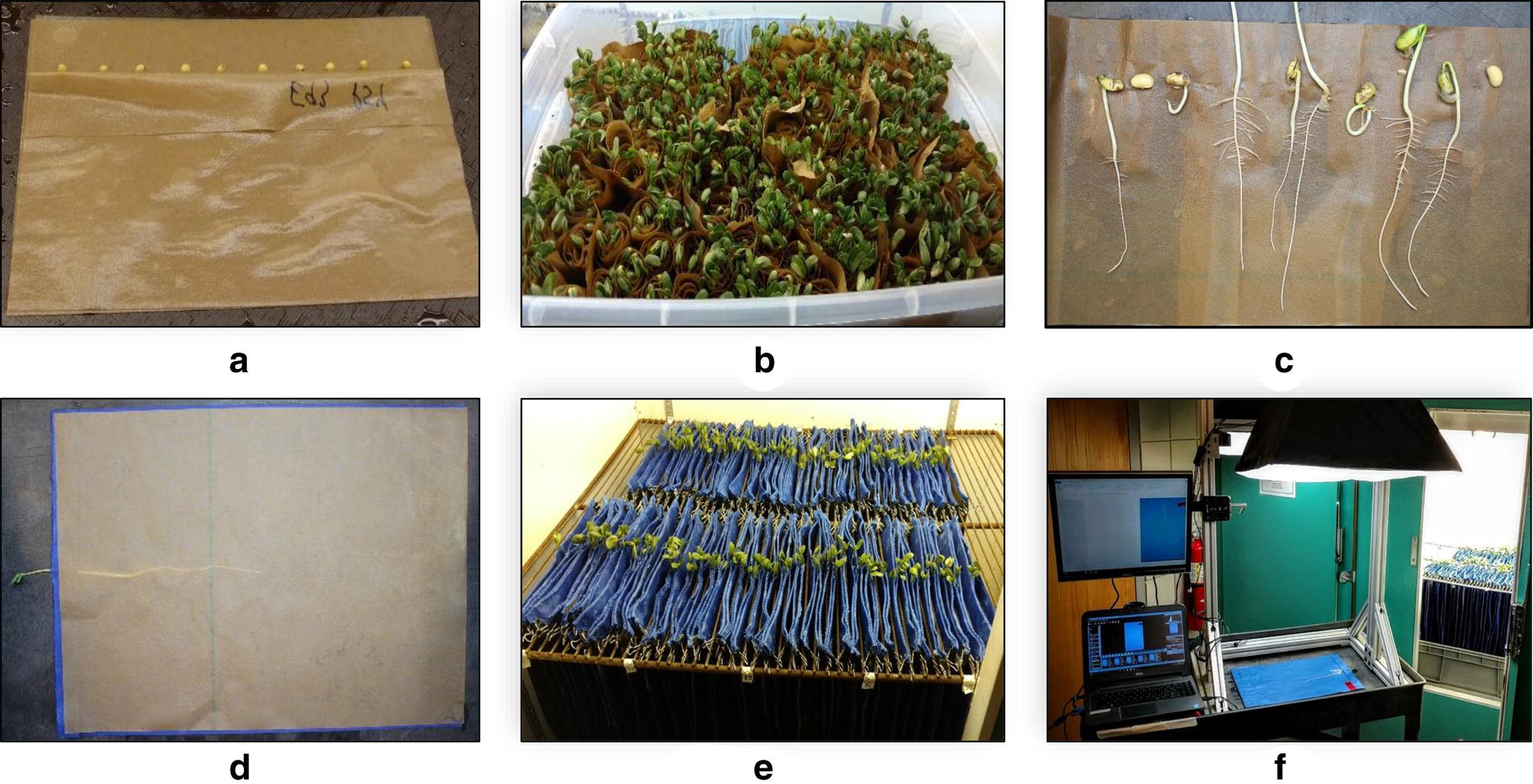
Root phenotyping platform. **a** 10 seeds per genotype rolled into germination paper. **b** Plants germinate in growth chamber and removed at 5 days. **c** Two representative seedlings are selected from each roll for transplantation onto labeled moist blue germination paper. **d** Single, transplanted seedlings are covered with moist brown germination paper and sandwiched together creating one experimental unit. **e** Experimental units are bound with binder clips, each placed between the metal rungs of a growth chamber with the bottom 2.5 cm submerged in water. **f** At 6* days*, 9 days and 12 days, experimental units are individually removed, split, imaged, automatically rotated, renamed via an image processing algorithm and saved to the server database, and replaced into the growth chamber

Motivated by previous research, we present a hardware system that is affordable and simple to construct, requiring few materials [95–98]. Seedlings are grown on the pouch-and-wick system [95] consisting of flat blue blotter germination paper (Anchor Paper Co., Minneapolis, MN), which creates high contrast with the yellow roots facilitating higher quality computer based root identification and segmentation [96]. A total of 4,088 seedlings were grown on blue blotter germination paper, suspended from the rungs of the shelving platform in a standard 1.75 m^2^ growth chamber during the course of the experiment (Fig. 1). The seedlings were phenotyped at three time points leading to 12,264 images that were a part of the overall experiment and a basis of software development for image processing and feature extraction.

The standard 175 cm by 100 cm growth chamber (Controlled Environments Ltd, Winnipeg, Canada) contains standard metal grate shelves (1.3 cm by 35 cm slots) which double as a support framework for suspending the germination paper above a water reservoir allowing for 2.5 cm of each paper unit to be submerged. The growth chamber could house up to 400 seedlings in 200 slots (corresponding to 228 seedlings m^2^). The growth chambers were set at 25 °C during a 16 h day, 22 °C for an 8 h night. Light intensity of the growth chambers was measured at 300 and 350 µmol photons m^−2^ s^−1^ respectively, as measured by a Li-250A light meter (Li-Cor Biosciences, Lincoln, NE, USA).

Ten seedlings were germinated using a paper roll assay (Fig. 1a, b) [99], of which two representatives of healthy seedlings were chosen at 5 days after germination and transplanted to the blue blotter germination system (Fig. 1c) [100] minimizing the variability associated with seed source of plant introduction lines [101] and the effect of poor or delayed germination [95]. When working with such diverse soybean plant introduction landraces seed uniformity and viability can be a hurdle. Specifically germination differences within genotypes was often substantial as displayed in Fig. 1c. To reduce variability across the experiment, 14 seedlings were grown and phenotyped for each genotype during the duration of this study. The apparatus and methodology of the germination paper roll assay is further presented in Additional file 1: Video S1 [102].

A blue blotter germination paper sheet cut to 30 cm × 45 cm with perforations made at 2.5 cm from top of the page was used in the experiment. The large sized germination paper allowed for undisturbed root growth for up to 12 days. Each sheet of blue paper was wetted and subsequently folded along the perforation to place one selected 5-day old seedling allowing for shoot penetration through the perforation. Thereafter, a wetted brown germination sheet was placed on top of the blue blotter paper and emerging radicle to isolate and adhere to each root to retain moisture (Fig. 1d). The thin brown paper is non-porous, preventing root penetration and allowing for easy removal prior to imaging. This procedure was repeated for the second seedling, after which the two seedlings of one genotype were affixed together using two binder clips. Each group of blue paper and brown germination paper combination housing two separated seedlings (hereon called, growth pouch unit) was suspended vertically via binder clips in slots between the labeled grates of the growth chamber with the lower 2.5 cm of blue paper submerged into water (Fig. 1e). Additional water was manually added to the reservoir as needed. Image capture began 6 days after germination, 1 day after transplanting, with consecutive images captured at 9 days and 12 days. The apparatus and methodology of the transplantation from brown to blue germination paper is further presented in Additional file 2: Video S2 [103].

### Imaging platform and protocol

**Fig. 2:**
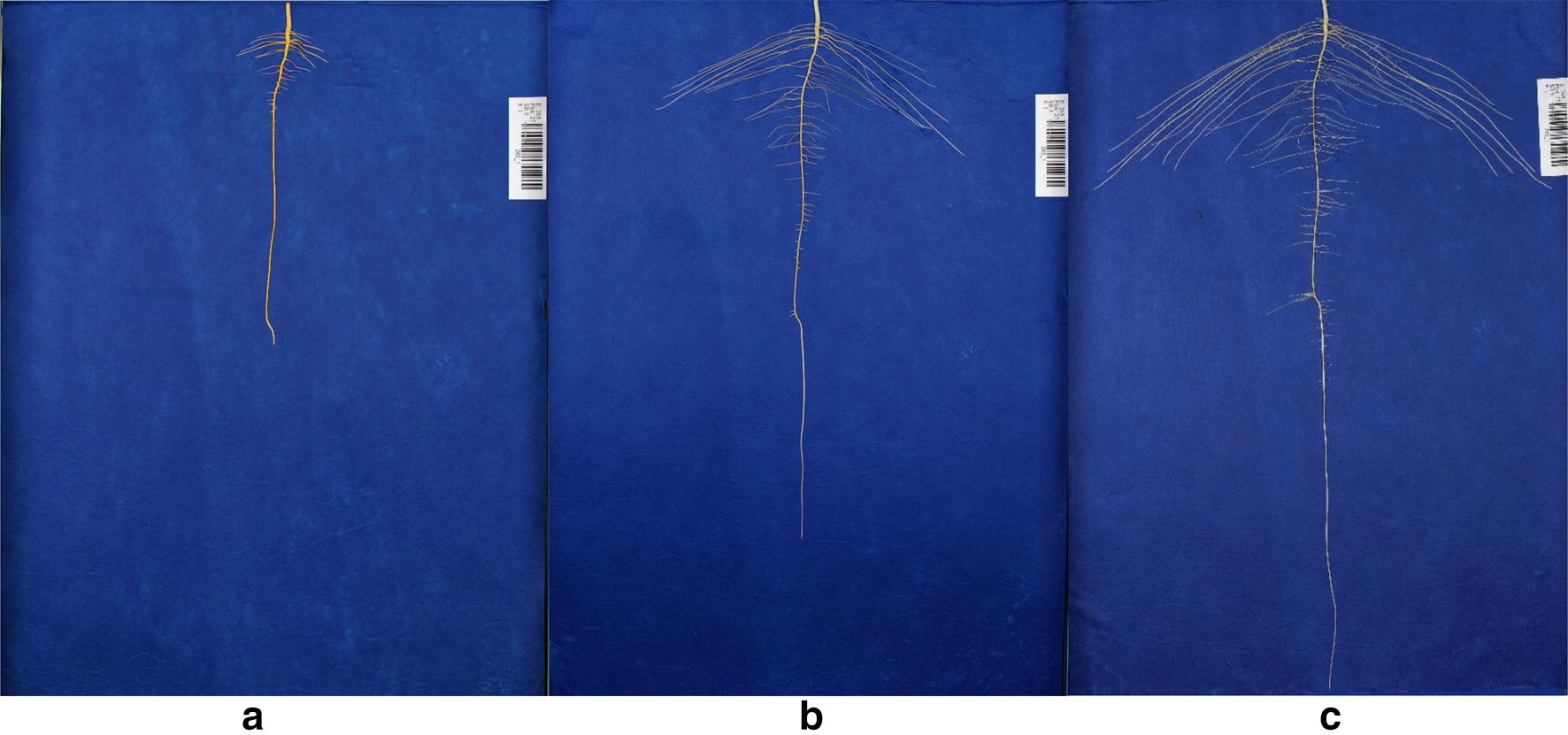
Time series growth of a single soybean plant with images taken at **a** 6 days, **b** 9 days and **c** 12 days after germination. Images were captured remotely via a laptop computer using software automating the image file renaming via the in-frame barcode. Smart Shooter 3 optimized the system’s throughput by renaming each image at acquisition using Object Character Recognition (OCR), reducing time and eliminating user input and human error. Image files were directly saved to a cloud-based database system. An additional computer monitor was affixed to the platform to facilitate manual inspection of captured images

The imaging platform consists of a utility cart, framework for camera mounting, and computer connectivity for image storage and file management. The imaging stage was fabricated using rugged, adjustable 80/20 aluminum T-slot extrusion (80/20 Inc., Columbia City, IN) (Fig. 1f) to provide a customizable, rigid structural framework including camera and light mounting to provide consistent image quality. Sensors included an 18 megapixel Canon Rebel T5i digital SLR camera (Lens: EF-S 18–55 mm f/3.5–5.6 IS II) (Canon USA, Inc, Melville, NY) mounted to a gimbal tripod head affixed to a T-slot extrusion crossbeam 60 cm directly above the imaging stage with the camera’s frame set to a consistent position at each imaging day. Additionally, the camera was positioned at a sufficient distance to capture the maximum length of a root at 12 days, as identified in preliminary experiments. The camera was set at a consistent white balance, focal length and maximum resolution to ensure high image quality (100 pixels per cm). The USB cable connected the camera to computer allowing for direct image transfer and live view of the imaging stage. To provide consistent illumination, two softbox photography lights (with four bulbs: 70 watts, 5500 K CFL) (Neewer; Shenzen, China) extending out from the stage at a height of 90 cm from the cart top base were directed at the imaging stage from opposite sides. The imaging platform was constructed on an Uline utility cart (Uline, Pleasant Prairie, WI), creating a compact mobile imaging station. The phenotyping platform consisted of off-the-shelf material and the total cost (excluding cameras and laptop) was less than $200. The remote capture software, Smart Shooter 3 [104] on a Dell Latitude E7470 laptop (Dell, Round Rock, TX), was used for a live view of the stage followed by triggering the camera for image capture. Plastic labels were affixed to each paper which included a unique barcode for each plant (Fig. 2).

Images were captured remotely via laptop computer using software automating the image file renaming via the in-frame barcode and current date. Smart Shooter 3 optimized the system’s throughput by renaming each image at acquisition using Object Character Recognition (OCR), reducing time and eliminating user input labor and human error [104]. Image files were directly saved to a cloud-based database system. An additional computer monitor was affixed to the platform to facilitate manual inspection of captured images. A list of system components can be found in Additional file 3: Table S1.

To capture images, each growth pouch unit was removed individually from the growth chamber grates, binder clips were removed, and blue paper and seedling combination were separated (Additional file 4: Video S3) [105]. Individual seedlings were placed on a 30 cm × 50 cm blue acrylic sheet for easier handling. Brown germination paper top sheet was removed and slight user adjustment of some roots was required to ensure that the computer algorithm could detect differences between multiple roots converging in parallel. A thin stainless steel laboratory spatula was used to lift and separate two side-by-side growing roots with an effort to reduce the movement and thus morphological change of the root and eliminate root damage. Root images were captured using a fixed digital SLR camera and a laptop computer as seen in Additional file 4: Video S3 [105]. After imaging, the growth pouch unit was reassembled and placed back into the growth chamber for the time series imaging pipeline. Seedling RSA analysis was conducted using a revised version of ARIA software (Table 1) [80]. To image approximately 300 seedlings, 3 to 5 h were needed dependent on number and experience of technical staff and growth stage of the seedling. The handling and imaging steps exposed the root to light up to 3 min.

**Table 1:** Root system architecture (RSA) traits captured by ARIA 2.0 software

Trait name	Symbol	Unit	Trait description
Total root length	TRL	cm	Cumulative length of all the roots in centimeters
Primary root length	PRL	cm	Length of the Primary root in centimeters
Lateral root length	LRL	cm	Cumulative length of all lateral roots in centimeters
Mean lateral root length	MSL	cm	Mean length of all lateral roots in centimeters
TRLUpper	TRLUpper	cm	Total root length of the upper one third
TRLLower	TRLLower	cm	Total root length of the lower two third
Perimeter	PER	cm	Total number of network pixels connected to a background pixel
Depth	DEP	cm	The maximum vertical distance reached by the root system
Width	WID	cm	The maximum horizontal width of the whole RSA
Diameter	DIA	cm	Diameter of the primary root
Lateral root branches	LRB	Count	Number of lateral root branches
Nodes of lateral roots	NLR	Count	Number of nodes of lateral roots
Independent root branches	IRB	Count	Number of independent lateral root branches
Lateral root tip	RTA	Count	Number of lateral root tips
Median	MED	Count	The median number of roots at all Y-location
MaximumR	MAX	Count	The maximum number of roots at all Y-location
Maximum number of roots	MNR	Count	The 84th percentile value of the sum of every row
Network area	NWA	Count	The number of pixels that are connected in the skeletonized image
Convex area	CVA	cm^2^	The area of the convex hull that encloses the entire root image
RhizoArea	RHZO	cm^2^	Length of 2 mm surrounding the TRL
TRArea	TRArea	cm^2^	Area of the RSA as observed in the 2D projected view
Primary root surface area	PRA	cm^2^	Surface area of the primary root
TRAUpper	TRAUpper	cm^2^	Total root area of the upper one third
TRALower	TRALower	cm^2^	Total root area of the lower two third
Volume	VOL	cm^3^	Volume of the primary root
Lateral root branching angle	LBA	Angle	Lateral root branching angle near the primary root node
Lateral root angles	LRA	Angle	Root angles along the extent of all lateral roots
Lateral root tip angle	RTA	Angle	Root angle at lateral root tips
Width/depth ratio	WDR	Ratio	The ratio of the maximum width to depth
Solidity	SOL	Ratio	The fraction equal to the network area divided by the convex area
Bushiness	BSH	Ratio	The ratio of the maximum to the median number of roots
Length distribution	LED	Ratio	TRLUpper/TRLower
LRL by PRL	LSLPL	Ratio	Number of the Lateral root per unit length of the Primary root
Center of mass	COM	Ratio	Center of gravity of the root/Depth
Center of point	COP	Ratio	Absolute center of the root regardless of root length/Depth
Center of mass (Top)	CMT	Ratio	Center of gravity of the top 1/3 of the root (Top)/Depth
Center of mass (Mid)	CMM	Ratio	Center of gravity of the middle 1/3 root (Middle)/Depth
Center of mass (Bottom)	CMB	Ratio	Center of gravity of the bottom 1/3 root (Bottom)/Depth
Center of point (Top)	CPT	Ratio	Absolute center of the root regardless of root length (Top)/Depth
Center of point (Mid)	CPM	Ratio	Absolute center of the root regardless of length (Middle)/Depth
Center of point (Bottom)	CPB	Ratio	Absolute center of the root regardless of root length (Bottom)/Depth

### Image processing

**Fig. 3:**
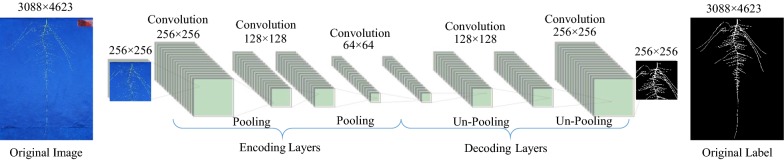
Convolutional Auto-Encoder with 32 feature maps of size 3 × 3 for each layer with two pooling layers of size 2 × 2 that were deployed for downsampling

Our platform was constructed to allow for post-capture automation, eliminating the requirement of image cropping and other image-preprocessing steps. An enhancement over the original release, ARIA 2.0 automatically detects the primary root allowing automated batch processing requiring minimal user input. In addition, ARIA 2.0 provides heuristic, k-means and convolution neural network (CNN) based segmentation functionality. The color segmentation method is based on a heuristic approach on the HSV (hue, saturation, value) color space (Additional file 5: Video S4). ARIA 2.0 provides a graphic user interface allowing for an optional quality check, manual adjustment and subsequent identification of problematic images. In our experience, the heuristic color segmentation was not successful for all images in a batch due to subtle differences caused by light reflection from infrequent over saturation of the blue germination paper. A ML approach was implemented to overcome the constraint of problematic images while providing full automation. The CNN based segmentation was built on a convolutional auto-encoder (CAE) architecture and implemented via MATLAB 2018b Deep Learning Toolbox (MathWorks, Inc., Natick, MA). CAE have been used as a robust method to segment features of object from a complex and cluttered background [89, 107]. To train our auto-encoder, we utilized the manual color segmentation method to generate a training data set. A set of randomly selected ~ 2450 images (20% of the dataset, including problematic and non-problematic images) across the three time points, were segmented and used to train the CAE (Fig. 3). The encoder segment of the final network architecture comprises three convolutional layers (32 feature maps of size 3 × 3 for each layer). In addition, two pooling layers of size 2 × 2 were deployed for downsampling the features and reducing the computational load. The Rectified Linear Unit (ReLU) function was used as the activation function. The learning rate was initialized with 0.001 using Adam optimization. Training was performed using a total of ~ 2100 samples with an additional 100 randomly selected validation samples, and testing was conducted on 250 random samples. We trained the model using a NVIDIA Tesla K20 installed on the CyEnce computing cluster at Iowa State University. Using a combination of binary cross entropy and Jaccard loss as loss function, the model was validated by fivefold cross-validation resulting in F1 score and IOU of 0.8824 and 0.8725, respectively. To further validate the model, a subset of 298 images segmented by both the heuristic and CAE methods resulted in a mean correlation of 0.91 across 23 ARIA 2.0 extracted root traits (Additional file 3: Table S2).

### Analysis

The original release of ARIA software measures length, surface area, volume and was validated through correlation with WinRHIZO Pro 9.0 (Regent Instruments, Quebec, Canada) [80]. Updated validations were conducted using ImageJ (https://imagej.nih.gov/ij/) and GiARoots [84] software. To assess broad sense heritability, 115 soybean genotypes belonging to soybean maturity group II with fourteen replications (genotypes were randomly assigned to the growth chamber) were grown in growth chambers and RSA traits were measured. Outliers that fell outside the interquartile range were identified for each trait for each genotype and were eliminated prior to calculating best linear unbiased predictors (BLUPs). The model used (Eq. 1) where y_ik_ is the response variable of the ith genotype at the kth block (i.e., growth chamber used), μ is the total mean, g_i_ is the genetic effect of the ith genotype, b_k_ is the block effect, and e_ik_, is a random error following N(0, σ^2^_e_). All factors were considered random effects. Broad sense heritability was calculated on an entry-mean basis using Eq. 2, where σ^2^_g_ is the genotypic variance, n is the number of replications = 14. Tukey’s Honest Significance Difference (HSD) groupings were calculated from the experimental data (alpha = 0.05, rep = 14 per genotype) where q = the relevant critical value of the studentized range statistic and n* is the number of scores used in calculating the group means of interest using the HSD.test function of the agricolae package in R (Eq. 3). Genetic coefficient of variation (CV_G_) for each trait was calculated using Eq. 4. Pearson’s correlation coefficients between traits were calculated using the ‘stats’ package in R. A Kolmogorov–Smirnov test, a nonparametric test of continuous probability distributions, was used to test statistical differences in directionality on root branching angle at each of the three time points after germination.

1 $$y_{ik} = u + g_{i} + b_{k} + e_{ik}$$

2 $$H^{2} = \frac{{\sigma_{g}^{2} }}{{\sigma_{g}^{2} + \frac{{\sigma_{e}^{2} }}{n}}}$$

3 $${\text{Tukey's Honest Significant Difference}} = q \sqrt {\frac{MSE}{{n^{*} }}}$$

4 $$C{V_G} = \frac{{\sqrt {{V_G}} }}{{\overline {\text{X}} }}$$

### Calculating lateral root branch count and measurement of root angles

**Fig. 4:**
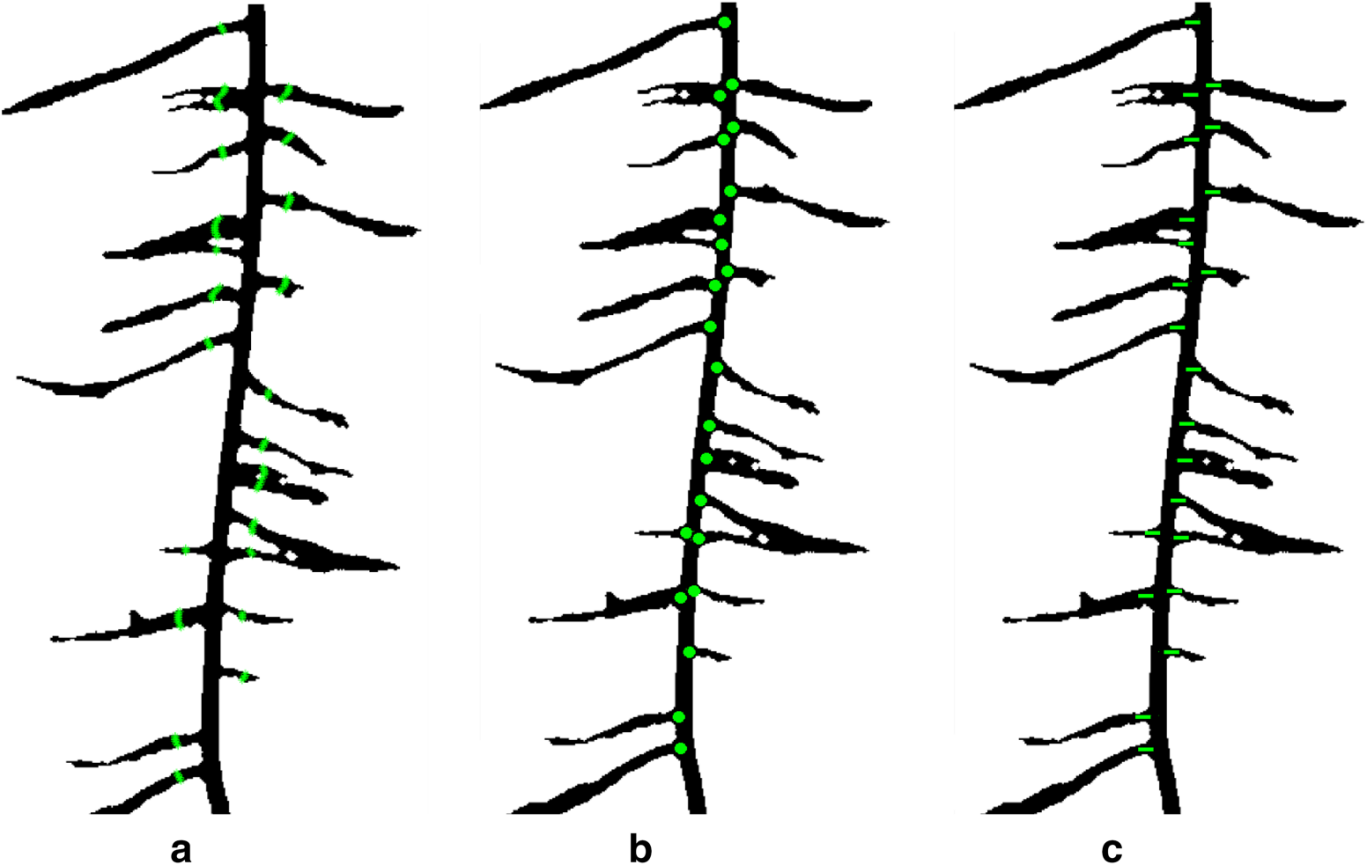
Lateral root branch count measured using three different methods **a** lateral root branch count (LRB), **b** count of nodes of lateral roots (NLR), and **c** independent lateral root branch count (IRB). The LRB method showed better correlation to ground truth data (R^2^ = 0.88 (LRB), R^2^ = 0.79 (NLR) and R^2^ = 0.76 (IRB))

RSA traits added to ARIA 2.0 include automated selection of the taproot, completely eliminating user-input, root measurement of root angles and lateral root branch count, which has been suggested as an important topological trait [1, 106]. Root branch counts were conducted using three different methods including: (1) lateral root branch count (LRB), nodes of lateral roots (NLR), and independent lateral root branches (IRB) (Fig. 4a–c) to determine the most informative and accurate way to study this trait. Lateral root branch count (LRB) was determined by first taking the skeletonized root in which the primary root is first removed. A sliding window with a five pixel width was moved across the root. The maximum number of individual root segments from each group (left or right of the primary root) were recorded. Nodes of lateral roots (NLR) were identified on the root skeleton using pixels that have more than two neighboring pixels (network analysis). The original black and white image is then dilated by 10 pixels, false or spurious branch points were identified and removed. The number of branch points after removing the false points was outputted.

**Fig. 5:**
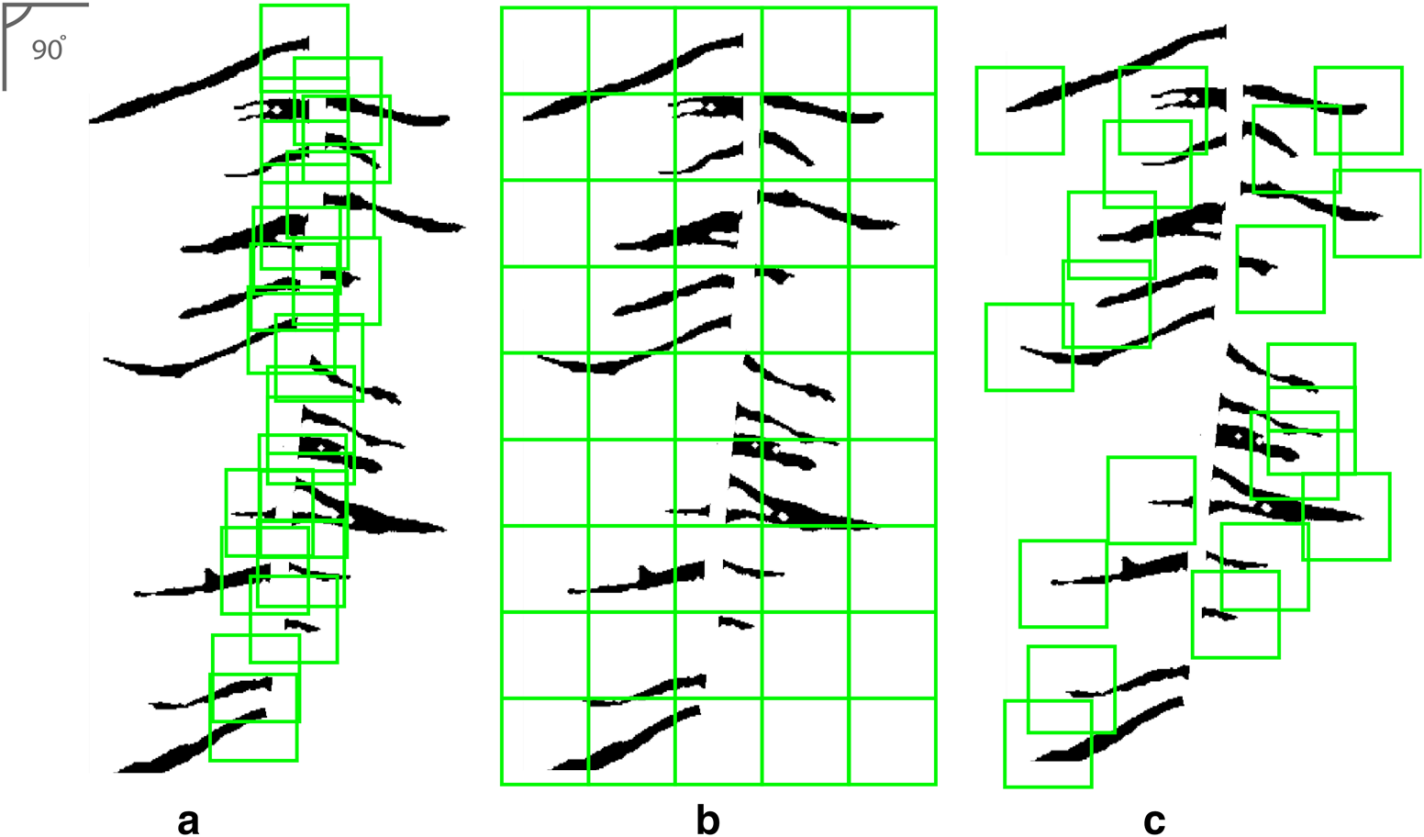
Three methods to identify lateral root angle including **a** lateral root branching angle (LBA), **b** lateral root angle along the entirety of each branch (LRA), and **c** lateral root tip angle (RTA)

Three algorithms of root branching angle were automatically quantified: (1) the lateral root branching angle (LBA), (2) lateral root angle (LRA), and (3) lateral root tip angle (RTA). The spatial distribution of the angles were shown with normalized vertical (soil) depth. The depth was normalized with the total root length. Measurement of root angles were generated within a 100 × 100 pixel window using a Fourier transform (to reduce noise), then a Hough transform algorithm was used to measure the angle of each segment (Fig. 5). The mean angle of each segment was placed into one of 45 bins of 2° increments from 0° to 90° (0° being vertical). For each algorithm, the lateral root angle of the highest frequency within each 100 × 100 pixel window was reported. ARIA 2.0 data output consisted of a tally of root segments for each algorithm allowing for simple visualization.

### Root shape classification

Two approaches were taken to extract root profiles from segmented root images, (1) mean boundary distance and (2) convex hull boundary. This dimension reduction processes distills images into simple numbers to allow for further phenotyping based applications. Fourier coefficients at multiple harmonics from 1 to 100 (Additional file 6: Figure S1) were used to create root shape profiles enabling the user to select which profile was most appropriate [107].

### Biomass weights

Shoot and root biomass weight was collected from plants at 12 days after being dried at 70 °C for 48 h. For each genotype, 100-seed weight (g) was recorded from the seed sources used for these experiments, and was obtained by counting random 100 seed for each genotype.

### Clustering algorithms (PCA and LDA)

Principal components analysis (PCA) and linear discriminant analysis (LDA) were used to visualize possible clusters of genotypes that form based on RSA traits. Linear discriminant analysis (LDA) finds a linear combination of RSA traits (explanatory variables) to discriminate between genotypes, the response variable. Principal components analysis (PCA) and LDA were performed using JMP ver. 13.1 (SAS Institute).

### Results

**Fig. 6:**
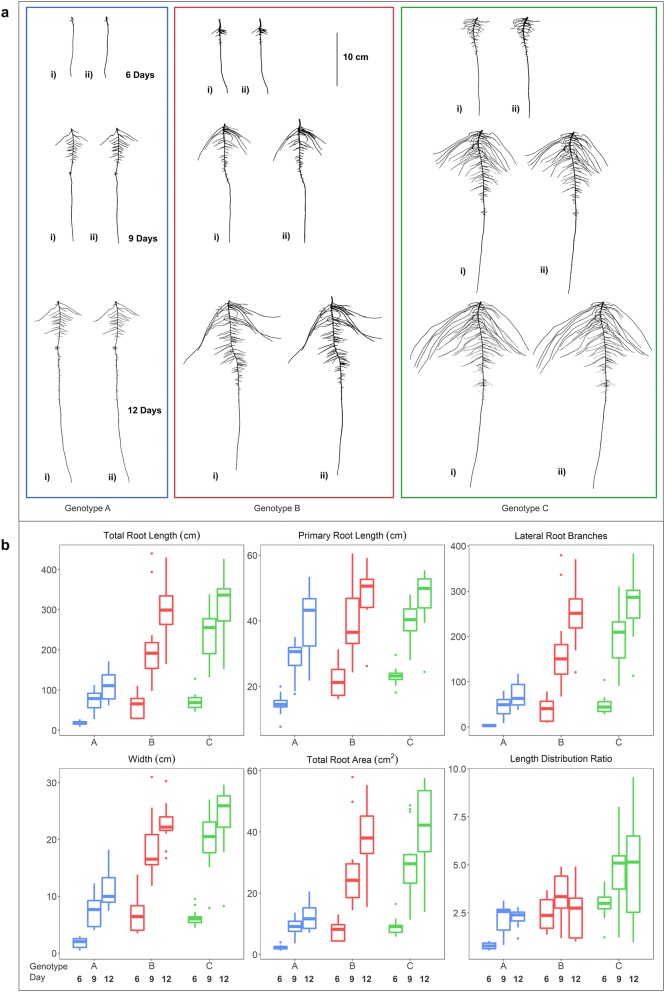
**a** Heuristic (i) and CAE (ii) based segmented root images of genotype A (blue), B (red) and C (green) at 6 (top), 9 (center) and 12 (bottom) days after germination. **b** Boxplot of displaying RSA traits of genotypes A (PI 417,138; blue), B (PI 643,146; red) and C (PI 479718B; green). TRL, PRL, LRB, WID, TRArea and LED were automatically calculated from the CAE segmented images by ARIA 2.0 (n = 14)

The end-to-end phenotyping pipeline consisted of the following main components: (1) a simple phenotyping platform capable of growing hundreds of plants, (2) automated image processing and curation system, (3) high fidelity image segmentation using both heuristic and ML approaches, and (4) root trait extraction software workflow and demonstrated through data analysis of 115 diverse soybean breeding lines. Using this pipeline 12,264 images were generated from 4088 plants. The image acquisition and processing rate varied depending on the number of technical persons available. This platform was used in single or multiple user modes, from individuals to teams of four, providing flexibility in time and labor management. Duration of image capture for 292 roots ranged from 3 to 5 h with throughput increased with younger roots and experienced technical staff. We present the capabilities of this root trait phenotyping pipeline to investigate the RSA diversity using test case samples consisting of diverse soybean genotypes from MG 2 using the heuristic segmentation approach and more in-depth study of three genotypes (PI 417,138, PI 643,146, PI 479718B) using the CAE segmentation approach for various RSA traits and visualize results for this diversity for the main traits (Fig. 6).

We evaluated the relationship between root traits and genotype descriptors including country of origin, stem termination and genetic diversity (Additional file 3: Table S3). A PCA plot was created using the genomic SNP data to further explore the associations between the continuous root trait data and discrete metadata (Additional file 6: Figure S2). Genotype was a significant source of variation for each trait (Additional file 3: Table S4). Heritability estimates increased concurrently with additional replicates and were highest for root and shoot dry weights (0.99) (Additional file 6: Figure S3).

### Validation of measurements

We reevaluated the updated ARIA framework by conducting manual benchmark validation. The fidelity of the measured traits depends on the accurate count of the pixels on the images, which was validated through primary root length measurements reported by ARIA 2.0 and confirmed manually with ImageJ software (R^2^ = 0.999) (Additional file 6: Figure S4). Furthermore, there was high correlation between traits extracted from a set of 300 heuristically segmented root images using GiARoots [84] software and ARIA 2.0 ranging between R^2^ = 0.659 (BSH) to R^2^ = 0.998 (CVA) with a mean of R^2^ = 0.916 (Additional file 3: Table S5).

ARIA 2.0 software was used to compare three methods of counting lateral root branches. The LRB method showed better correlation to ground truth data (R^2^ = 0.88 (LRB), R^2^ = 0.79 (NLR) and R^2^ = 0.76 (IRB)). Correlation between ImageJ manually extracted angles and ARIA 2.0 measurement of root tip angles was conducted to validate our approach after outliers were removed with a R^2^ = 0.9025 (Additional file 6: Figure S5). Minimal root angle diversity was noted among the three genotypes (Additional file 6: Figure S6). A Kolmogorov–Smirnov test was used to detect statistical differences in directionality on root branching angle at each of the three time points. When comparing between genotypes, significant differences (p < 0.05) were seen at 6 days between genotypes A and B and genotypes A and C (Additional file 3: Table S6).

Using root images segmented with the CAE approach, three genotypes PI 417,138 (genotype A), PI 643,146 (genotype B), PI 479718B (genotype C)) were further assessed. These three genotypes displayed diversity in several biologically relevant RSA traits (TRL, PRL, LRB, WID, TRArea, and LED (length distribution)) (Additional file 6: Figure S7). Genotype A was distinct from B and C for each of the six traits except for LED (a root trait based on a ratio of TRLUpper/TRLLower) it did not differ from genotype B and 9 days and 12 days (Table 2). Genotypes B and C differed from each other at 6 days for LED, at 9 days for all six traits, and at 12 days for TRL, WID, TRArea and LED. Shoot dry weight and root dry weight BLUPs were highly correlated (R = 0.86; genotypes = 115). The majority of RSA traits had significant correlation with both shoot and root dry weight (Additional file 3: Table S7).

**Table 2:** RSA trait mean values obtained from CAE segemented images, Tukey’s Honest Significant Difference (HSD) test groupings and growth rate day^−1^ for genotypes A, B and C for TRL (total root length), PRL (primary root length), LRB (lateral root branching count), WID (root width), Area (total root area) and LED (length distribution, total root length of the upper 1/3 of the root image divided by the total root length in the lower 2/3 of the root image

Day	TRL (cm)			PRL (cm)			LRB			WID (cm)			TRArea (cm^2^)			LED		
	6	9	12	6	9	12	6	9	12	6	9	12	6	9	12	6	9	12
Genotype A																		
BLUP	33.5	121	184.9	16.9	32.1	42.7	18.7	51.4	84.4	3.4	10	13.8	3.1	9.3	14.6	1.1	2.0	2.1
HSD grouping	b	c	c	b	c	b	c	c	b	b	c	c	b	c	c	c	b	b
Growth day^−1^		29.2	21.3		5.1	3.5		10.9	11.0		2.2	1.3		2.1	1.8		0.3	0.0
Genotype B																		
BLUP	67.2	216.2	342.9	21.3	36.2	48.1	33.5	71.5	121.3	6.5	16.8	22.3	6.5	19.5	27.6	1.9	2.5	2.2
HSD grouping	a	b	b	a	b	a	b	b	a	a	b	b	a	b	b	b	b	b
Growth day^−1^		49.7	42.2		5.0	4.0		12.7	16.6		3.4	1.8		4.3	2.7		0.2	‒ 0.1
Genotype C																		
BLUP	76.1	255.2	393.3	21.5	37.2	47.6	39.1	75.8	103.8	5.9	18.3	25	6.8	23.6	36	2.1	3.4	3.1
HSD grouping	a	a	a	a	a	a	a	a	a	a	a	a	a	a	a	a	a	a
Growth day^−1^		59.7	46.0		5.2	3.5		12.2	9.3		4.1	2.2		5.6	4.1		0.4	‒ 0.1

### Root shape classification

**Fig. 7:**
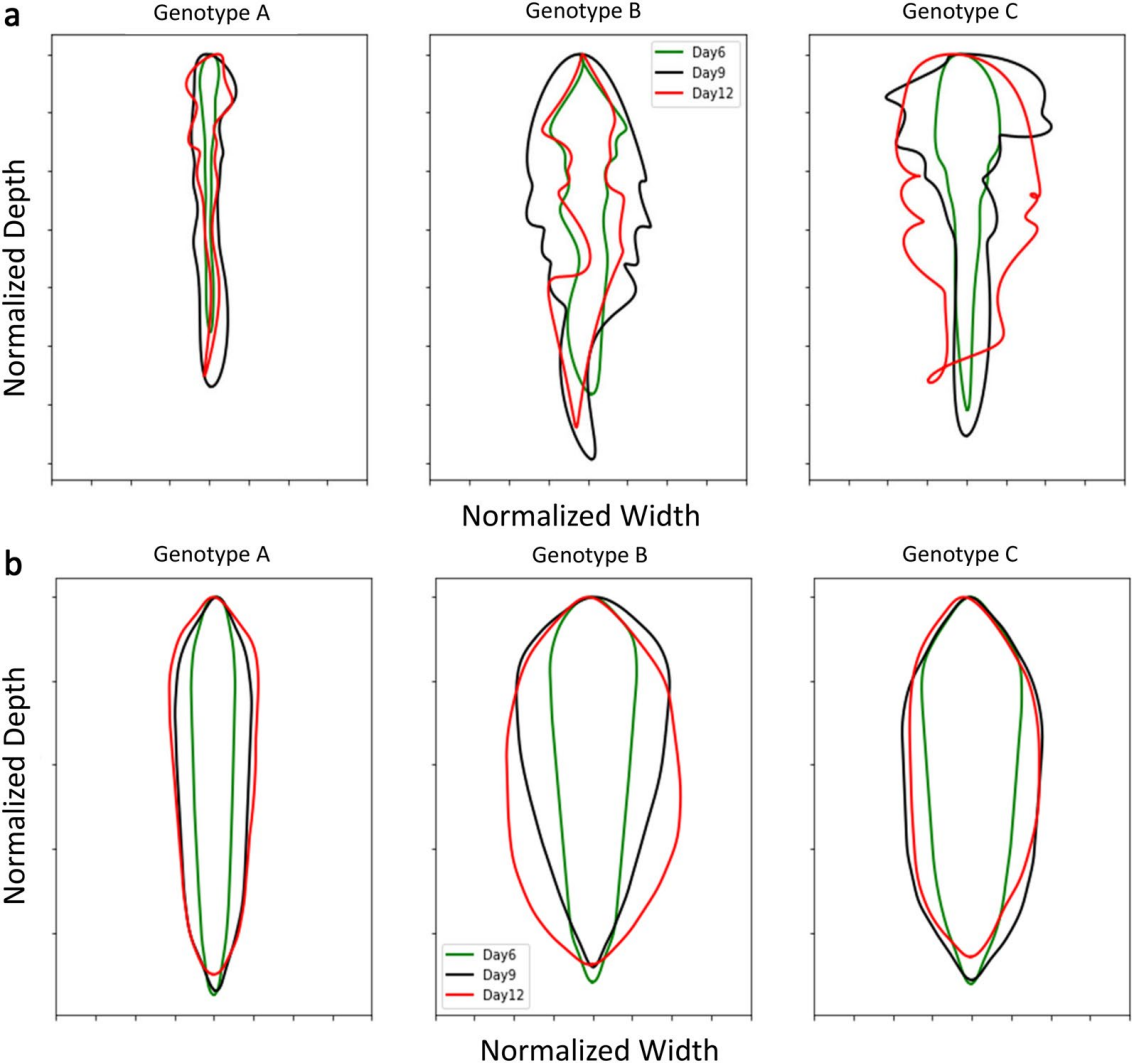
**a** Mean boundary based on five harmonic Fourier descriptors of genotypes A (PI 417,138) (left), B (PI 643,146) (middle), and C (PI 479718B) (right) at 6 days (green), 9 days (black), 12 days (red). **b** Convex hull boundary of root shape developed from Fourier analysis (five harmonic descriptors) of the three genotypes at 6 days (green), 9 days (black), 12 days (red) (n = 14)

Root profiles were extracted from images and using Fourier coefficients were expanded into a shape spectrum. Figure 7a displays pseudo-outline (i.e., mean boundary) of normalized values from three genotypes to highlight root shape variation. A similar approach was taken using convex hull area (CVArea) as an input (Fig. 7b). Mean boundary and convex hull boundary analyses identified interesting divergences in root shape between genotypes. PCA and LDA were used to evaluate the contributions of RSA traits between genotypes at multiple growth stages using the output of ARIA 2.0 at 6 days, 9 days, and 12 days. LDA revealed distinct clustering patterns, where observations of three genotypes at three separate time points after germination created nine groupings (Additional file 6: Figure S8a). The PCA based on root shape defining traits at 9 days and 12 days creates three clusters while the results at 6 days are not as definitive (Additional file 6: Figure S8b).

### Discussion

**Fig. 8:**
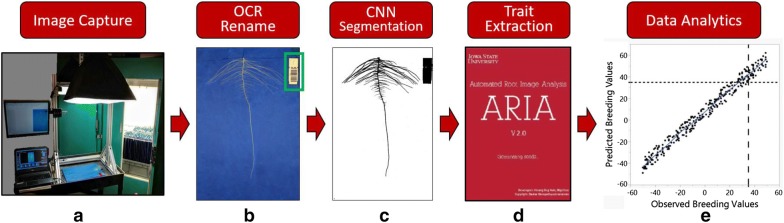
Proposed root phenotyping pipeline. **a** Root phenotyping platform. **a** Image stage fabricated from aluminum, softbox lights, Canon T5i, laptop computer and, LCD monitor to evaluate images quality and image database. **b** Software scans and renames image automatically using barcode. **c** CNN framework identifies and segments root from background. **d** ARIA 2.0 extracts RSA traits from root images. **e** Data analytics (genomic selection, GWAS) are performed

In this paper, we describe end-to-end hardware and software solutions for a soybean root phenotyping pipeline (Fig. 8). This phenotyping platform provides a non-destructive evaluation pipeline with high repeatability, ease of use and scalability, capacity for hundreds genotypes in a short period of time at a lowered cost and a level of automation that will meet the needs of plant breeding. The seedling growth apparatus requires minimal supplies, expense, and experience to set up. ARIA 2.0 graphic based user interface is simple, straight-forward and builds on previous work [80]. Improvements to ARIA include additional functionality including more RSA traits, including root shape and multiple segmentation approaches. Despite the original release of ARIA being an alternative option to established programs such as WinRHIZO Pro 9.0, the application was limited to binary images and batch processed root images in a semi-automatic manner requiring user-input to identify the taproot. ARIA 2.0 was developed to address deficiencies and build additional functionality, customization and automation. The high-throughput root imaging system and fully autonomous batch processing of thousands of images with ARIA 2.0 allow for an automated imaging pipeline. In an effort to capture the essence of a root, we integrated a holistic approach which identified root shape as a trait in the phenotyping framework using Fourier transformations. We created a color segmentation package that is user-customizable for specific applications. The outcome served as a training set which fed into our ML segmentation application, reducing time through automated handling of problematic images. ARIA 2.0 software is an open-source application with simple installation, requiring no other software to operate. Multiple methods of root branching angle and counts were added to the original software’s extracted root traits. The low barrier (cost and technical expertise) to entry and rapid image capture and data processing time make this phenotyping platform suitable for large scans of diverse genetic material, genetic mapping studies, RSA trait studies and selection strategies in breeding.

### Low-cost imaging platform

As research in roots has expanded, technologies such as high resolution digital cameras and computational power have increased along with specialized technicians. While we limit the presentation of blue paper for imaging background, our preliminary studies were done using gellan gum, hydroponics and brown paper cigar rolls (data not presented). Pouch-and-wick systems using blue germination paper have been routinely used to analyze and record root growth in previous studies [25, 95–98]*.* The correlation to root growth between different systems or media such as germination paper, hydroponic or gellan-gum and soil suggests there are differences that affect the growth of the roots; however, since all genotypes in this study were tested under the same system we were able to make comparisons between genotypes. The blue germination paper approach was deemed most suitable as it allowed simultaneous growing and imaging of hundreds of plants nondestructively in a time series manner with a minimal person-hour requirement. For soybean, at 12 days post germination, the taproot of a limited number of genotypes outgrew the blue paper system; longer lengths of paper can be used if the intent is to perform post 12 day imaging.

The imaging platform’s efficiency was reduced by root convergence as previously identified by Dupuy et al. [97]. Slight user adjustment of some roots was required to ensure that the computer vision algorithm could detect differences between multiple roots converging in parallel. Our preliminary studies concluded that convergence was inconsistent between root and genotypes and could reduce TRL up to 18% (data not shown) due to the software’s inability to identify individual roots out of a mass. Therefore, our protocol was developed with minimal manual adjustment to reduce convergence. Removal of the technician adjustment would decrease the duration of image acquisition. Further experiments are needed to solve the issue of convergence without adding a root processing step.

While our system shows promise for a time-series root trait data collection, continual work is needed to expand the ability to phenotype large number of genotypes in a wider time-series manner and also establishing controlled environment and field grown trait relationship.

### ML based image preprocessing and image analysis

Plant phenotyping can vary in number of experimental treatments and genotypes and thus degrees of complexity [108]. While alternate root image analysis software tools exist, our platform implements additional RSA traits alongside automated machine learning segmentation to make it feasible for use with large data sets. In a step towards full automation from image acquisition through analysis, user input is minimized, removing the interaction between the user and individual root images (such as determining anchor points with ARIA (original release) [80] or SmartRoot [66]). The system is capable of scaling to both large root systems and large quantities of root systems. Alternative software, such as Root System Analyzer, are capable of using image sequences to track growth. However, even the fully automated system requires substantial user intervention (data not presented) and is more amenable for smaller datasets. Traditional image segmentation methods are not generalizable, as users’ needs to trial and error to identify the best segmentation model and model input parameter (heuristically). More importantly, it is our experience that while some (traditional) segmentation models work on a good fraction of images, no segmentation model works on (nearly) all the images. The end-user then has to either make a sub-optimal choice or perform exhaustive quality control. This is where ML approaches like the CAE model become attractive. With a small amount of annotated data, they can be specifically trained for a specialized application with minimal subsequent user input. We present the end-to-end pipeline we included both hardware and software advances for root trait imaging and analytics without exclusively focusing on ARIA 2.0 improvement. ML has become a critical tool to improve analysis and quantification of data in plant phenomics [56]. Until recently, the use of ML in root phenomics has been limited to root tip identification [91, 109] and data prediction [92]. Using a CAE for image preprocessing, is a new approach in root phenomics, and overcomes current challenges in image preprocessing.

### RSA trait measurements

The ideal root architecture is dependent on breeding objectives as desired architecture may be determined by crop, environment, fertility, and water availability however are often not well described [1]. Using computer vision tools, the creation and collection of RSA traits is nearly endless. What is important however, is to collect biologically relevant traits. The system described in this paper delivers as much information to the user as possible so that the user can then determine the biological usefulness of each trait as per the objectives of their studies.

**Fig. 9:**
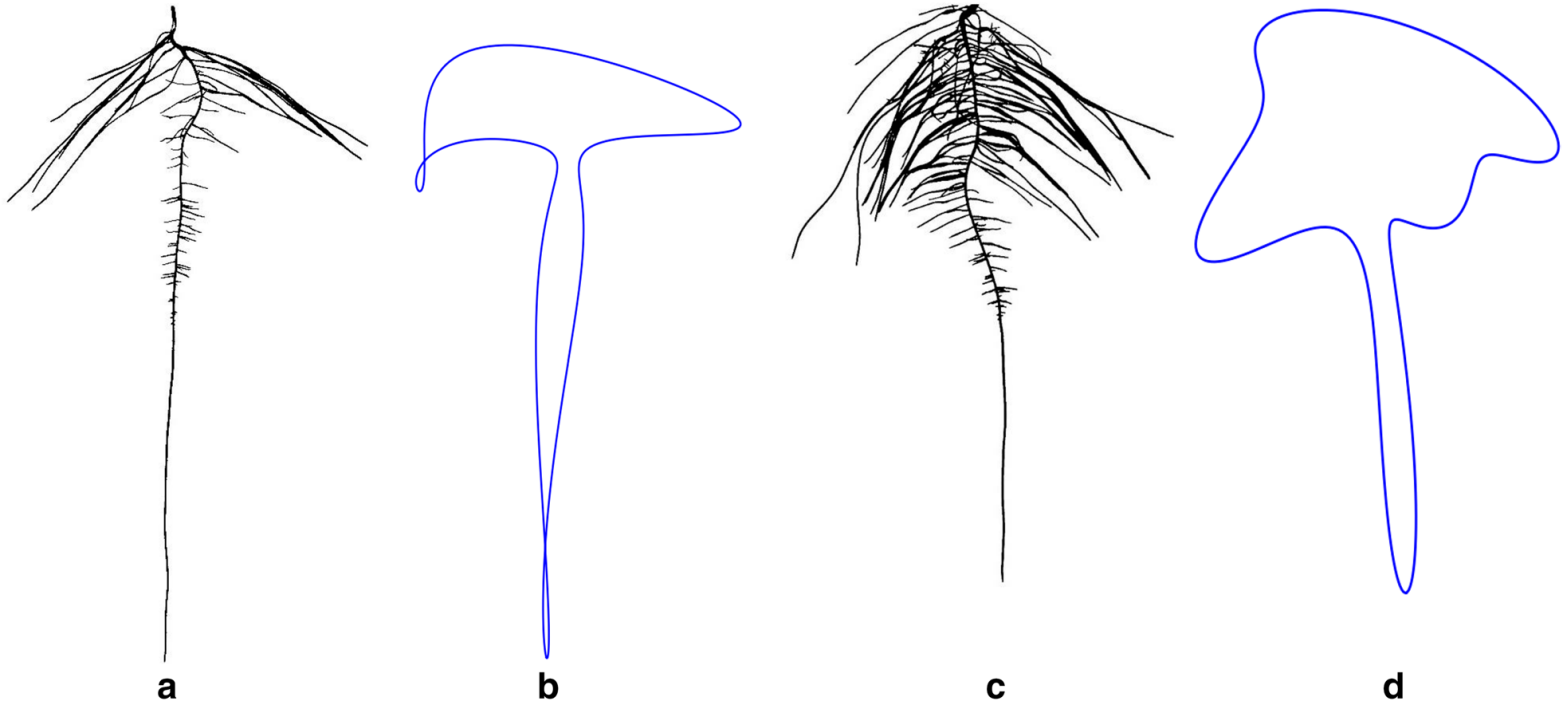
Root shape profiles based on elliptical Fourier transformation (EFT). Example genotypes of **a** LG05-4832, **b** EFT derived root outline of LG05-4832 (n = 14), **c** PI 594457A and, **d** EFT derived root outline of PI 594457A (n = 14) at 9 days

ARIA 2.0 software was used to compare three improved methods of counting first-order lateral root branches, which were validated using manual assessments of 68 random plants from the three time points (6 days, 9 days, and 12 days). Automated identification of second-order lateral roots could be an addition of future versions of ARIA. Lateral root branch number (LRB) had the strongest correlation to the ground truth results. NLR and IRB often overestimated the number of roots compared to manual counts likely due to the misidentification of pixel spurs. Misidentification of pixels spurs, an erroneous grouping of pixels on the boundary of the blue paper which is a product of the segmentation process, as roots resulted in false positives. Multiple roots growing together in parallel were liable to be counted as one, as color-based image segmentation was unable to isolate individual roots. One particular improvement to ARIA is the measurement of root angles, with respect to the direction of gravity, taken in three locations, near the primary root, at the root tip and throughout the root system as a whole. Previous studies have shown that root angle in rice [39, 110], chickpea (*Cicer arietinum* L.) [111] and sorghum (*Sorghum bicolor* L.) [112] is correlated to drought tolerance and root depth. The correlation between root angle and drought tolerance was identified in rhizotrons [85] because water-deficient Arabidopsis roots grow at a steeper angle than well-watered roots [85, 113, 114]. Aside from root count, primary root volume and surface area can also be found in ARIA 2.0. Thick roots have been shown to penetrate deeper through soil-layers [115, 116]. Thin, fibrous roots have shown plasticity in response to drought. Large, thick roots act as a conduit pipe and serve a purpose in anchorage however, it is the fine secondary and tertiary roots that make up the vast network of absorbing area. Plants that optimize root absorption area while minimizing biological cost are desirable [36]. The aforementioned traits can be identified using the ARIA 2.0 seedling phenotyping pipeline, demonstrating the relevance of ML and computer vision based software for the study of RSA traits. Furthermore, the presented approach can also be useful in learning or describing new traits, and studies on the growth and development in a time-series manner. Unlike above ground traits, at this time root systems do not have a well characterized growth or stages. An understanding of stages and processes is integral to translating root development into mathematical growth models, which can help develop more efficient plants. Compelling visual differences for root shape were uncovered, exhibited by heuristically segmented root images of LG05-4832 and PI 594457A in Fig. 9. Aided by Fourier descriptors, soybean canopy shapes have been previously described [107, 117]. Using a similar approach, we observed that these methods were sufficient to draw root outlines. This system was designed to maximize data acquisition and to reduce environmental differences with minimal errors. Therefore, this pipeline was effective in root trait studies to identify most diverse genotypes from a germplasm set; however further improvements are needed to enable complex organism interaction studies and field grown genotype roots sample assessments. For example, the image based root phenotyping methods still need additional technological refinement and advancement to integrate microorganism-root interaction phenotyping and studies [11, 12, 118, 119].

Controlled environment studies bring advantages of scale and data noise reduction along with cost efficiency gains. Since selection of root traits in row crops is one of the major challenge for breeding programs, we envision a two-step approach: (1) using a germplasm and pre-breeding step in controlled environment screening of root traits similar to this study and previous studies [25, 81, 96, 98] to assemble a smaller collection of accessions or experimental lines for further testing, and (2) field screening for root traits through direct and indirect selection. Since, for most row crop breeding programs, the ultimate goal is increase seed yield, indirect selection for yield traits will need identification of root traits with high heritability and high genetic correlation between root traits and seed yield. While advances in phenotyping and data analytics of above ground traits is gaining exponentially, similar advances in root traits are lacking due to complexity of phenotyping organs below ground and a spatio-temporal scale. Therefore, studies that build on expanding the inference scope of root trait are needed with connectivity with yield performance. While our system shows promise for a time-series root trait data collection, continual work is needed to expand the ability phenotype large number of genotypes in a wider time-series manner and also establishing controlled environment and field grown trait relationship. The novelty of our work is developing an end-to-end phenotyping system, and integration of ML based batch image pre-processing and root trait feature extraction. We envision that approach (1) will help in determining the genetic variation for root traits and thereby influencing selection differential factor of response to selection. This work provides insights on root trait diversity from a large collection of the USDA germplasm bank, and one of the largest such report on soybean root studies.

## Conclusions

This report describes innovation through the development of an affordable end-to-end phenotyping system of hundreds of plants, and integration of ML based batch image pre-processing and root trait feature extraction. We have developed a phenotyping pipeline that integrates image capture, image processing and image analysis of growing plant roots in controlled conditions providing a high-throughput, cost effective platform yielding biologically relevant time-series data on root growth and development. The outcome of hardware and software solutions provided a high quantity, cost-effective, efficient, repeatable seedling root phenotyping platform incorporating time series growth capture, and a computer vision based ARIA 2.0 integrated with ML based image preprocessing step. Additionally, we demonstrated the potential of the pipeline to capture RSA trait diversity on three selected soybean genotypes, which can be expanded to larger genotype set. HTP methods together with phenomics and data analytics [120] will give researchers the tools needed to decipher the genetics of RSA trait expression to realize the potential of root driven breeding. Further work is needed to develop methods for 3D reconstruction, as well as methodologies to link and reduce the gap between controlled and field experiment root studies. We envision that approach (1) will help in determining the genetic variation for root traits and thereby influencing selection differential factor of response to selection and prescriptive plant breeding [121] .

## Supplementary information

**Additional file 1: Video S1.** Initiation of germination rolls.

**Additional file 2: Video S2.** Transplanting soybean seedlings at five days after germination.

**Additional file 3: Table S1.** A list of imaging system components. **Table S2.** Validation correlations between 23 RSA traits extracted from ARIA 2.0 using 298 heuristically segmented and CAE segmented root images. **Table S3.** RSA traits at 6d, 9d and 12d when grouped into country of origin, growth habit type and diversity of genetic background. **Table S4.** Descriptive statistics for 6 root and shoot traits of 115 maturity group II genotypes of soybean at 6d, 9d and 12d, obtained from BLUP values for each genotype using heuristically segmented images. **Table S5.** Validation correlation between GiARoot software and ARIA 2.0. **Table S6.** Minimal root angle diversity among the three genotypes. A Kolmogorov-Smirnov test was used to detect p-value statistical differences in directionality on root branching angle at each of the three time points. **Table S7.** Correlations between plant dry weight taken at 12d and root traits at 9d for 115 maturity group II genotypes of soybean.

**Additional file 4: Video S3.** Soybean seedling root imaging.

**Additional file 5: Video S4.** Color segmentation method of root images using a heuristic approach in MATLAB based on the HSV (hue, saturation, value) color space.

**Additional file 6: Figure S1.** Root shape profiles derived from elliptical Fourier transformations (EFT) at multiple harmonics (n = 2 to n = 20). **Figure S2.** PCA plots based on genomic SNP data to further explore the associations between (a) country of origin, (b) growth habit, and (c) genetic diversity (elite, diverse, landrace). **Figure S3.** Six RSA traits displaying the increase in broad-sense heritability (H2) with each replicate tested (n=14). **Figure S4.** Validation of primary root length (PRL) using SmartRoot in ImageJ. ** Figure S5.** Correlation between manual and ARIA 2.0 root angles. Correlation resulted in an R2 value of 0.9025. Any root angles calculated by ARIA 2.0 as being less than 10 degrees were considered as being outliers due to a result of very small root segments. **Figure S6.** Root angles of genotype A (PI 417138; blue), B (PI 643146; red) and C (PI 479718B; green) at 6d (left), 9d (center) and 12d (right) generated from heuristically segmented images. The top row relates to LBA; middle row, LRA; and bottom row is RTA (all expressed as a percentage of total). 0° is the direction of the gravity vector. **Figure S7.** Overall phenotypic differentiation of three example soybean genotypes: A (PI 417138; blue), B (PI 643146; red) and C (PI 479718B; green) for TRL (total root length), PRL (primary root length), WID (root width), convex area (CVA) LRB (lateral root branching count), VOL (primary root volume), LRA (lateral root branching angle, LED (length distribution, total root length of the upper 1/3 of the root image divided by the total root length in the lower 2/3 of the root image), RHZO (rhizosphere area), WDR (width to depth ratio), Root_weight (dry root weight at 12 days after germination), Shoot_weight (dry shoot weight at 12 days after germination). For every trait each datum is reflective of one root image (therefore there are 14 data points per genotype). The data lines reflect the mean of the data points for respective genotype. Heuristically segmented images were used in this analysis. **Figure S8.** Dimension reduction analysis. (a) Linear discriminant analysis works as a dimensionality reduction algorithm, is shown using 38 RSA traits to cluster genotype A (PI 417138; blue), B (PI 643146; red) and C (PI 479718B; green) at 6 (triangle), 9 (plus) and 12 (diamond) after germination (n=14). (b) Principal components analysis of the three genotypes at 6, 9 and 12 days after germination. The shaded area enclose 90% of each genotype’s data points (n=14; generated from heuristic segmented images).

## Data Availability

The ARIA 2.0 code is freely available at the address: https://bitbucket.org/baskargroup/aria2/src/master/. Analysis code is freely available at the address: https://github.com/mighster/ARIA2.0. Raw images and/or segmented masks are available upon request.

## Abbreviations

ARIA: automatic root imaging analysis
BLUP: best linear unbiased prediction
CAE: convolutional auto-encoder
CNN: convolutional neural network
CV_G_: genetic coefficient of variation
DL: deep learning
HSD: Tukey’s honest significant difference
HSV: hue, saturation, value
HTP: high-throughput phenotyping
LDA: linear discriminant analysis
ML: machine learning
OCR: object character recognition
PCA: principal components analysis
RSA: root system architecture
