# Supplementary material for: Computer vision and machine learning enabled soybean root phenotyping pipeline
Source: Plant Methods. 2020 Jan 23;16:5. doi: 10.1186/s13007-019-0550-5 (PMC6977263; doi:10.1186/s13007-019-0550-5)
Supplement: Supplementary file 3 — Additional file 3: Table S1. A list of imaging system components. Table S2. Validation correlations between 23 RSA traits extracted from ARIA 2.0 using 298 heuristically segmented and CAE segmented root images. Table S3. RSA traits at 6d, 9d and 12d when grouped into country of origin, growth habit type and diversity of genetic background. Table S4. Descriptive statistics for 6 root and shoot traits of 115 maturity group II genotypes of soybean at 6d, 9d and 12d, obtained from BLUP values for each genotype using heuristically segmented images. Table S5. Validation correlation between GiARoot software and ARIA 2.0. Table S6. Minimal root angle diversity among the three genotypes. A Kolmogorov-Smirnov test was used to detect p-value statistical differences in directionality on root branching angle at each of the three time points. Table S7. Correlations between plant dry weight taken at 12d and root traits at 9d for 115 maturity group II genotypes of soybean. [file 13007_2019_550_MOESM3_ESM.docx]

| **System Component** | **Quantity** | **Description** |
| --- | --- | --- |
| Straight wall container | 4 | Plastic storage container (190.5mm height x 812.8mm length x 381mm width; W.W. Grainger, Lake Forest, IL ) |
| Binder clips | 600 | Small spring tight metal binder clip (19.05mm; Staples, Framingham, MA) |
| Blue blotter germination paper | 300 sheets | Steel blue seed germination blotter (482.6mm x 304.8mm; Anchor Paper, Minneapolis, MN) |
| Brown germination paper | 300 sheets | Brown germination paper (482.6mm x 304.8mm; Anchor Paper Co., Minneapolis, MN) |
| Plastic labels | 300 | Plant tag Slip 'N Lock thermal transfer perforated loop labels (25.4mm x 177.8mm; A.M. Leonard, Piqua, OH) |
| Fabric cutter | 1 | Rotary cutter with 45mm perforated wheel (Fiskars, Helsinki, Finland) |
| Plastic container | 2 | Plastic storage container (266.7mm height x 692.15mm length x 431.8mm width; Rubbermaid, Atlanta, GA ) |
| Forceps | 4 | Stainless steel forceps (114.3mm; W.W. Grainger, Lake Forest, IL ) |
| Data logger | 1 | Track-It RH/Temp Data Logger w/display (Monarch Instument, Amherst, NH) |
| Acrylic sheet | 4 | Transparent acrylic sheet (482.6mm x 304.8mm; Lowes, Mooresville, NC) |
| Canon EOS Rebel T5i | 1 | 18.0MP DSLR camera, 18-55mm IS STM lens (Canon, Tokyo, Japan) |
| Oben BE-117 ball head mount | 1 | Ball head with quick mount (Gradus Group LLC, New York, NY) |
| Laptop | 1 | Dell Latitude 7470 (Dell, Round Rock, TX) |
| USB cable | 1 | USB-A to mini USB cable |
| Aluminum frame | 1 | 80/20 T-slotted extrusion (80/20 Inc. Columbia City, IN) |
| Softbox light | 2 | 700W 5500k photography studio softbox (B&H Photo, New York, NY) |

Table S1: A list of imaging system components.

Table S2: Validation correlations between 23 RSA traits extracted from ARIA 2.0 using 298 heuristically segmented and CAE segmented root images.

| **Trait** | **R** |
| --- | --- |
| TRL | 0.97 |
| PRL | 0.98 |
| SRL | 0.97 |
| MED | 0.98 |
| MAX | 0.96 |
| LED | 0.75 |
| PER | 0.98 |
| DIA | 0.71 |
| VOL | 0.78 |
| Area | 0.86 |
| TRLUpper | 0.94 |
| TRLLower | 0.86 |
| CVA | 0.97 |
| DEP | 0.99 |
| WID | 0.98 |
| NWA | 0.96 |
| RHZO | 0.98 |
| LRB | 0.85 |
| NLR | 0.80 |
| IRB | 0.84 |
| Area | 0.92 |
| TRAUpper | 0.92 |
| TRALower | 0.90 |
| Mean | 0.91 |

Table S3: RSA traits at 6d, 9d and 12d when grouped into country of origin, growth habit type and diversity of genetic background.

|  |  |  |  |  |  |  |  |  |  |  |  |  |  | **Root** | **Shoot** | **Seed** |
| --- | --- | --- | --- | --- | --- | --- | --- | --- | --- | --- | --- | --- | --- | --- | --- | --- |
|  | **Day** | **n** | **TRL** | **TRL_GR** | **PRL** | **VOL** | **WID** | **WDR** | **CVA** | **RHZO** | **LRB** | **LRA** | **LED** | **Weight** | **Weight** | **Weight** |
| China | 6 | 78 | 70.5 | 23.5 | 21.0 | 89.6 | 6.0 | 0.31 | 65.8 | 1161 | 38.1 | 85.4 | 1.73 | - | - | - |
| Japan | 6 | 8 | 58.6 | 19.5 | 19.9 | 81.5 | 5.2 | 0.27 | 55.4 | 967 | 32.7 | 85.3 | 1.54 | - | - | - |
| Korea | 6 | 3 | 75.7 | 25.2 | 21.4 | 111.1 | 6.9 | 0.34 | 77.7 | 1245 | 37.8 | 85.2 | 1.99 | - | - | - |
| Other | 6 | 9 | 67.2 | 22.4 | 21.0 | 87.1 | 5.6 | 0.29 | 59.9 | 1107 | 38.8 | 85.4 | 1.69 | - | - | - |
| Russia | 6 | 11 | 73.0 | 24.3 | 21.1 | 101.6 | 6.2 | 0.32 | 68.0 | 1212 | 37.6 | 85.3 | 1.80 | - | - | - |
| USA | 6 | 6 | 69.6 | 23.2 | 21.2 | 98.5 | 6.2 | 0.32 | 68.1 | 1149 | 38.2 | 85.2 | 1.88 | - | - | - |
| China | 9 | 78 | 220.9 | 49.4 | 35.9 | 156.4 | 16.3 | 0.50 | 299.0 | 3791 | 72.8 | 86.9 | 2.66 | - | - | - |
| Japan | 9 | 8 | 177.6 | 40.0 | 34.4 | 136.1 | 13.8 | 0.45 | 244.6 | 3027 | 65.7 | 86.5 | 2.34 | - | - | - |
| Korea | 9 | 3 | 238.2 | 56.5 | 37.9 | 216.1 | 18.2 | 0.52 | 352.5 | 4057 | 74.6 | 87.6 | 3.19 | - | - | - |
| Other | 9 | 9 | 214.1 | 48.7 | 36.0 | 156.5 | 15.9 | 0.48 | 290.2 | 3651 | 77.3 | 86.8 | 2.56 | - | - | - |
| Russia | 9 | 11 | 231.9 | 53.1 | 36.6 | 171.3 | 17.5 | 0.52 | 328.4 | 4019 | 72.1 | 87.3 | 2.71 | - | - | - |
| USA | 9 | 6 | 211.2 | 47.3 | 37.4 | 169.1 | 16.2 | 0.48 | 311.5 | 3617 | 75.7 | 86.7 | 2.62 | - | - | - |
| China | 12 | 78 | 340.8 | 40.3 | 47.6 | 213.4 | 21.3 | 0.51 | 512.1 | 5720 | 104.4 | 89.0 | 2.89 | 0.09 | 0.21 | 13.7 |
| Japan | 12 | 8 | 277.5 | 31.3 | 45.9 | 207.9 | 18.3 | 0.47 | 424.6 | 4559 | 99.3 | 88.8 | 2.59 | 0.08 | 0.20 | 12.6 |
| Korea | 12 | 3 | 389.8 | 45.5 | 49.7 | 258.2 | 24.3 | 0.54 | 627.3 | 6666 | 117.8 | 89.1 | 2.82 | 0.11 | 0.27 | 19.2 |
| Other | 12 | 9 | 329.5 | 38.8 | 47.8 | 217.7 | 20.9 | 0.50 | 502.9 | 5573 | 113.0 | 89.0 | 2.65 | 0.08 | 0.21 | 13.1 |
| Russia | 12 | 11 | 378.2 | 47.1 | 48.7 | 253.9 | 23.7 | 0.55 | 588.8 | 6437 | 109.1 | 89.1 | 2.96 | 0.10 | 0.23 | 14.6 |
| USA | 12 | 6 | 319.2 | 36.3 | 49.2 | 240.9 | 20.4 | 0.48 | 504.8 | 5332 | 113.4 | 89.1 | 2.66 | 0.10 | 0.25 | 16.5 |
|  |  |  |  |  |  |  |  |  |  |  |  |  |  |  |  |  |
| Determinate | 6 | 25 | 72.2 | 24.1 | 21.2 | 95.3 | 6.2 | 0.32 | 67.2 | 1181 | 38.4 | 85.6 | 1.83 | - | - | - |
| Indeterminate | 6 | 71 | 67.8 | 22.6 | 20.8 | 88.6 | 5.9 | 0.30 | 64.2 | 1127 | 36.8 | 85.2 | 1.68 | - | - | - |
| Semi-determinate | 6 | 19 | 73.5 | 24.5 | 21.1 | 94.6 | 6.1 | 0.31 | 66.5 | 1194 | 40.2 | 85.7 | 1.78 | - | - | - |
| Determinate | 9 | 25 | 229.2 | 51.8 | 36.1 | 167.9 | 16.5 | 0.50 | 306.3 | 3913 | 74.3 | 86.6 | 2.68 | - | - | - |
| Indeterminate | 9 | 71 | 212.4 | 47.6 | 36.0 | 152.3 | 16.1 | 0.49 | 297.0 | 3669 | 71.5 | 86.9 | 2.62 | - | - | - |
| Semi-determinate | 9 | 19 | 226.3 | 51.4 | 35.9 | 170.2 | 16.3 | 0.50 | 299.2 | 3817 | 75.3 | 87.2 | 2.69 | - | - | - |
| Determinate | 12 | 25 | 358.5 | 41.5 | 47.5 | 225.7 | 21.4 | 0.51 | 513.8 | 5968 | 107.9 | 88.9 | 2.82 | 0.10 | 0.25 | 16.4 |
| Indeterminate | 12 | 71 | 329.2 | 39.5 | 48.0 | 216.8 | 21.2 | 0.51 | 516.0 | 5580 | 105.3 | 89.1 | 2.81 | 0.09 | 0.21 | 13.4 |
| Semi-determinate | 12 | 19 | 351.5 | 40.8 | 47.0 | 223.4 | 21.6 | 0.53 | 514.1 | 5798 | 105.7 | 89.1 | 2.98 | 0.09 | 0.22 | 12.7 |
|  |  |  |  |  |  |  |  |  |  |  |  |  |  |  |  |  |
| Diverse | 6 | 4 | 70.9 | 23.6 | 21.0 | 103.2 | 6.4 | 0.33 | 69.5 | 1165 | 38.9 | 85.3 | 1.87 | 0.11 | 0.25 | 16.0 |
| Elite | 6 | 2 | 67.1 | 22.4 | 21.6 | 89.2 | 5.8 | 0.29 | 65.3 | 1117 | 36.9 | 85.0 | 1.90 | 0.09 | 0.24 | 17.5 |
| Landrace | 6 | 109 | 69.7 | 23.2 | 20.9 | 90.6 | 6.0 | 0.31 | 65.1 | 1150 | 37.7 | 85.4 | 1.72 | 0.09 | 0.22 | 13.8 |
| Diverse | 9 | 4 | 216.1 | 47.9 | 37.6 | 180.4 | 16.8 | 0.49 | 325.7 | 3698 | 76.2 | 86.7 | 2.62 | 0.11 | 0.25 | 16.0 |
| Elite | 9 | 2 | 201.3 | 46.1 | 37.0 | 146.4 | 15.0 | 0.45 | 283.1 | 3454 | 74.8 | 86.6 | 2.63 | 0.09 | 0.24 | 17.5 |
| Landrace | 9 | 109 | 218.7 | 49.2 | 35.9 | 158.0 | 16.2 | 0.50 | 298.7 | 3754 | 72.6 | 86.9 | 2.65 | 0.09 | 0.22 | 13.8 |
| Diverse | 12 | 4 | 332.2 | 38.2 | 49.9 | 265.6 | 21.7 | 0.51 | 545.0 | 5580 | 114.5 | 89.2 | 2.74 | 0.11 | 0.25 | 16.0 |
| Elite | 12 | 2 | 293.3 | 32.4 | 47.9 | 191.6 | 17.6 | 0.43 | 424.5 | 4836 | 111.3 | 89.0 | 2.52 | 0.09 | 0.24 | 17.5 |
| Landrace | 12 | 109 | 340.4 | 40.4 | 47.7 | 218.7 | 21.4 | 0.51 | 515.8 | 5721 | 105.6 | 89.0 | 2.85 | 0.09 | 0.22 | 13.8 |

Table S4: Descriptive statistics for 6 root and shoot traits of 115 maturity group II genotypes of soybean at 6d, 9d and 12d, obtained from BLUP values for each genotype.

| **Day** | **Trait** | **TRL (cm)** | **PRL (cm)** | **LRB** | **WID (cm)** | **Area (cm^2^)** | **LED** | **Shoot (g)** | **Root (g)** |
| --- | --- | --- | --- | --- | --- | --- | --- | --- | --- |
| **6** | Mean | 68.79 | 20.84 | 37.45 | 5.91 | 6.06 | 1.72 |  |  |
|  | Median | 68.79 | 21.01 | 37.45 | 5.90 | 6.06 | 1.71 |  |  |
|  | Maximum | 217.69 | 37.16 | 108.00 | 22.35 | 23.68 | 2.05 |  |  |
|  | Std Deviation | 39.95 | 5.61 | 20.14 | 3.38 | 3.63 | 0.11 |  |  |
|  | ANOVA (Genotype) | *** | *** | *** | *** | *** | *** |  |  |
|  | Heritability | 94% | 89% | 88% | 90% | 94% | 80% |  |  |
|  | CVg | 22% | 8% | 19% | 21% | 22% |  |  |  |
| **9** | Mean | 213.01 | 34.90 | 70.91 | 15.77 | 18.28 | 2.50 |  |  |
|  | Median | 208.53 | 35.62 | 70.00 | 15.55 | 17.51 | 2.60 |  |  |
|  | Maximum | 545.16 | 78.31 | 330.00 | 33.03 | 49.53 | 4.08 |  |  |
|  | Std Deviation | 93.02 | 8.14 | 25.99 | 5.78 | 8.18 | 0.43 |  |  |
|  | ANOVA (Genotype) | *** | *** | *** | *** | *** | *** |  |  |
|  | Heritability | 93% | 82% | 86% | 93% | 93% | 69% |  |  |
|  | CVg | 20% | 7% | 14% | 19% | 22% |  |  |  |
| **12** | Mean | 333.86 | 45.64 | 102.98 | 20.66 | 27.29 | 2.63 | 0.22 | 0.09 |
|  | Median | 328.29 | 47.62 | 98.00 | 21.31 | 26.10 | 2.62 | 0.22 | 0.09 |
|  | Maximum | 1860.31 | 72.62 | 232.00 | 33.33 | 122.72 | 3.73 | 0.49 | 0.19 |
|  | Std Deviation | 130.25 | 9.89 | 39.70 | 6.04 | 11.40 | 0.35 | 0.07 | 0.03 |
|  | ANOVA (Genotype) | *** | *** | *** | *** | *** | *** | *** | *** |
|  | Heritability | 91% | 80% | 85% | 93% | 91% | 66% | 1.00 | 1.00 |
|  | CVg | 21% | 8% | 14% | 17% | 22% |  | 0.27 | 0.24 |
| * | < 0.05 |  |  |  |  |  |  |  |  |
| ** | < 0.01 |  |  |  |  |  |  |  |  |
| *** | < 0.001 |  |  |  |  |  |  |  |  |

Table S5: Validation correlation between GiARoot software and ARIA 2.0.

| **Trait** | **R^2^** |
| --- | --- |
| TRL | 0.996 |
| PER | 0.904 |
| DEP | 0.994 |
| WID | 0.945 |
| CVA | 0.998 |
| Area | 0.996 |
| WDR | 0.929 |
| SOL | 0.766 |
| BSH | 0.659 |
| LED | 0.974 |
| Mean | 0.916 |

Table S6. Root angle diversity among the three genotypes. A Kolmogorov-Smirnov test was used to detect p-value statistical differences in directionality on root branching angle at each of the three time points.

|  | **Genotype** | | |
| --- | --- | --- | --- |
| **Day** | **A** | **B** | **C** |
| **d6/d9** | 0.02202 | 0.04774 | 0.87495 |
| **d9/d12** | 0.06863 | 0.98005 | 0.72851 |
| **d6/d12** | 0.00004 | 0.06548 | 0.38231 |
|  |  |  |  |
|  | **Day** | | |
| **Genotype** | **d6** | **d9** | **d12** |
| **A/B** | 0.00625 | 0.06328 | 0.99997 |
| **B/C** | 0.78103 | 0.99128 | 0.85781 |
| **A/C** | 0.00008 | 0.16646 | 0.96162 |

Table S7: Correlations (r) between plant dry weight taken at 12d and root traits at 9d for 115 maturity group II genotypes of soybean

|  | **Dry Weight** | |
| --- | --- | --- |
| **Root Trait** | **Shoot** | **Root** |
| Shoot Weight | - | 0.86 |
| Root Weight | 0.86 | - |
| TRL | 0.61 | 0.76 |
| PRL | 0.41 | 0.48 |
| SRL | 0.61 | 0.75 |
| MSL | 0.38 | 0.51 |
| TRLUpper | 0.63 | 0.67 |
| TRLLower | 0.62 | 0.74 |
| NWA | 0.60 | 0.75 |
| PER | 0.51 | 0.67 |
| DEP | 0.35 | 0.42 |
| WID | 0.55 | 0.65 |
| DIA | 0.68 | 0.71 |
| LRB | 0.52 | 0.60 |
| NLR | 0.45 | 0.52 |
| IRB | 0.47 | 0.55 |
| MED | 0.48 | 0.57 |
| MAX | 0.57 | 0.73 |
| CVA | 0.57 | 0.66 |
| Rhzo | 0.56 | 0.72 |
| Area | 0.72 | 0.84 |
| PRA | 0.35 | 0.51 |
| TRAUpper | 0.72 | 0.82 |
| TRALower | 0.45 | 0.60 |
| VOL | 0.62 | 0.64 |
| WDR | -0.37 | -0.45 |
| SOL | -0.14 | -0.16 |
| BSH | 0.10 | 0.24 |
| LED | 0.19 | 0.20 |
| LSLPL | -0.04 | -0.02 |
