# Supplementary material for: Computer vision and machine learning enabled soybean root phenotyping pipeline
Source: Plant Methods. 2020 Jan 23;16:5. doi: 10.1186/s13007-019-0550-5 (PMC6977263; doi:10.1186/s13007-019-0550-5)
Supplement: Supplementary file 6 — Additional file 6: Figure S1. Root shape profiles derived from elliptical Fourier transformations (EFT) at multiple harmonics (n = 2 to n = 20). Figure S2. PCA plots based on genomic SNP data to further explore the associations between (a) country of origin, (b) growth habit, and (c) genetic diversity (elite, diverse, landrace). Figure S3. Six RSA traits displaying the increase in broad-sense heritability (H2) with each replicate tested (n=14). Figure S4. Validation of primary root length (PRL) using SmartRoot in ImageJ. Figure S5. Correlation between manual and ARIA 2.0 root angles. Correlation resulted in an R2 value of 0.9025. Any root angles calculated by ARIA 2.0 as being less than 10 degrees were considered as being outliers due to a result of very small root segments. Figure S6. Root angles of genotype A (PI 417138; blue), B (PI 643146; red) and C (PI 479718B; green) at 6d (left), 9d (center) and 12d (right) generated from heuristically segmented images. The top row relates to LBA; middle row, LRA; and bottom row is RTA (all expressed as a percentage of total). 0° is the direction of the gravity vector. Figure S7. Overall phenotypic differentiation of three example soybean genotypes: A (PI 417138; blue), B (PI 643146; red) and C (PI 479718B; green) for TRL (total root length), PRL (primary root length), WID (root width), convex area (CVA) LRB (lateral root branching count), VOL (primary root volume), LRA (lateral root branching angle, LED (length distribution, total root length of the upper 1/3 of the root image divided by the total root length in the lower 2/3 of the root image), RHZO (rhizosphere area), WDR (width to depth ratio), Root_weight (dry root weight at 12 days after germination), Shoot_weight (dry shoot weight at 12 days after germination). For every trait each datum is reflective of one root image (therefore there are 14 data points per genotype). The data lines reflect the mean of the data points for respective genotype. Heuristically segmented [file 13007_2019_550_MOESM6_ESM.pdf]

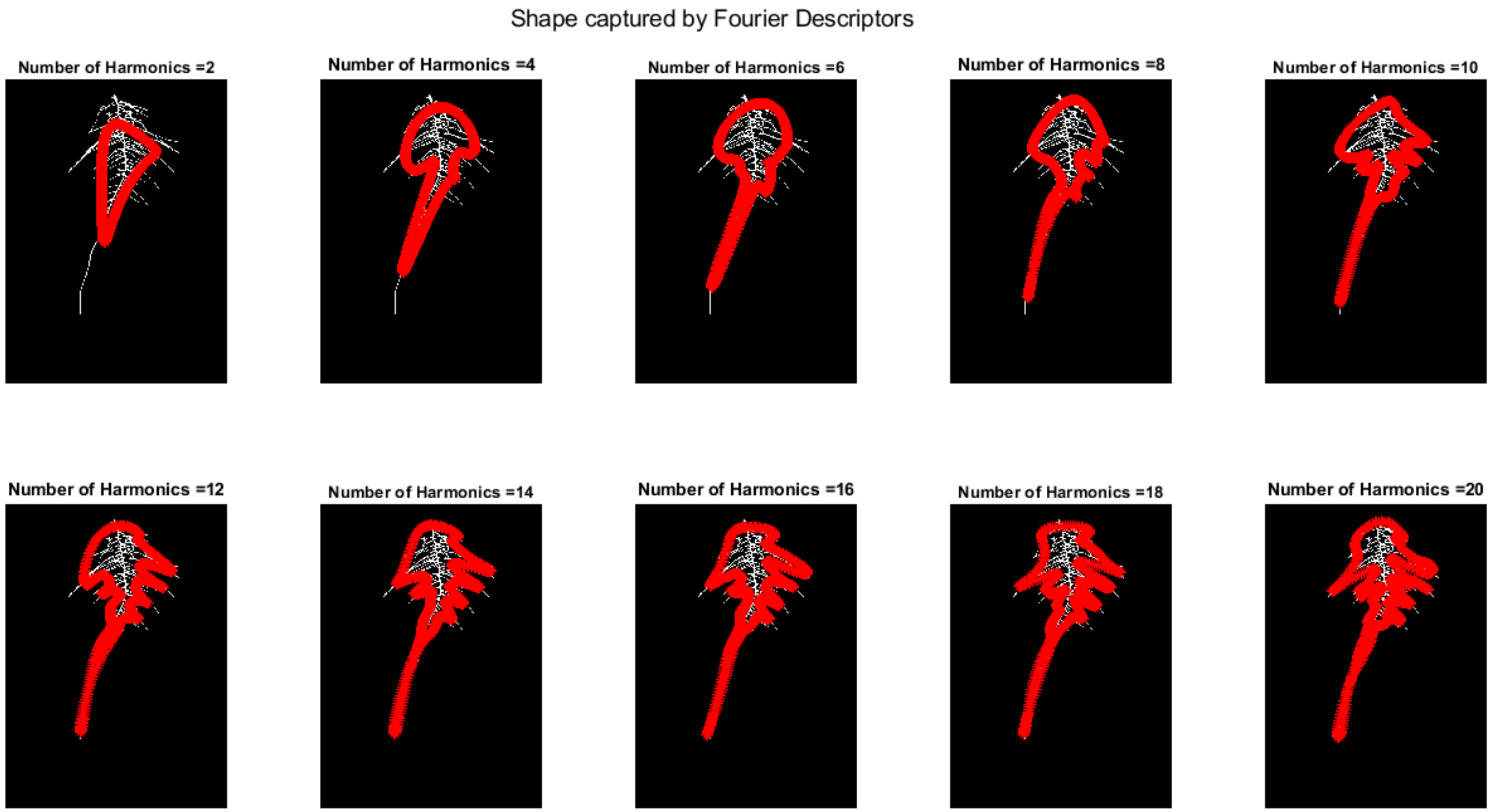

Supplementary Figure 1

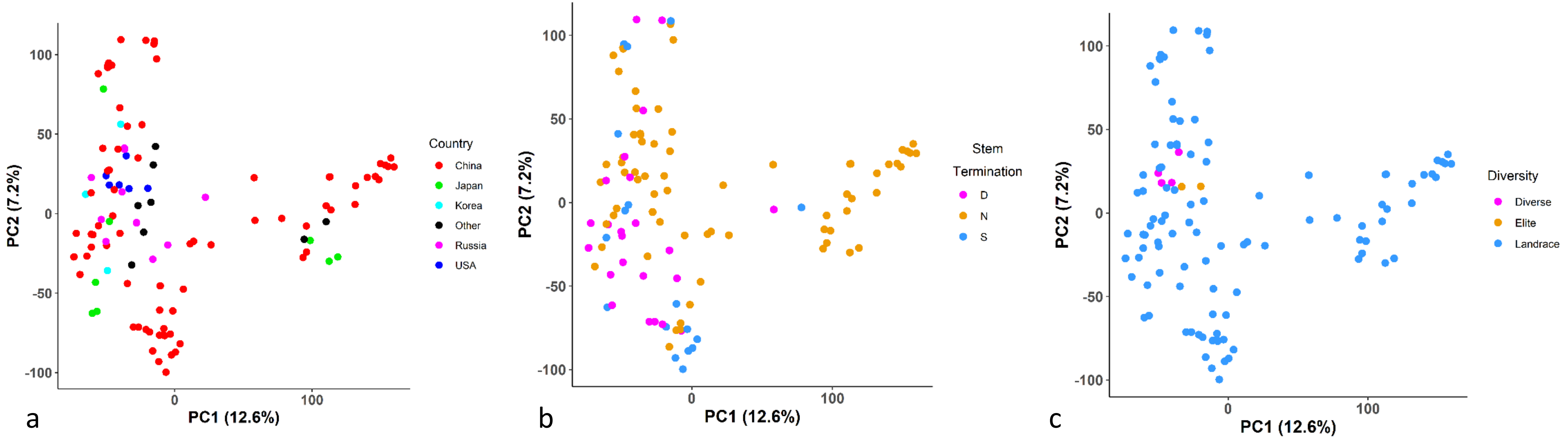

Supplementary Figure 2 a,b, c

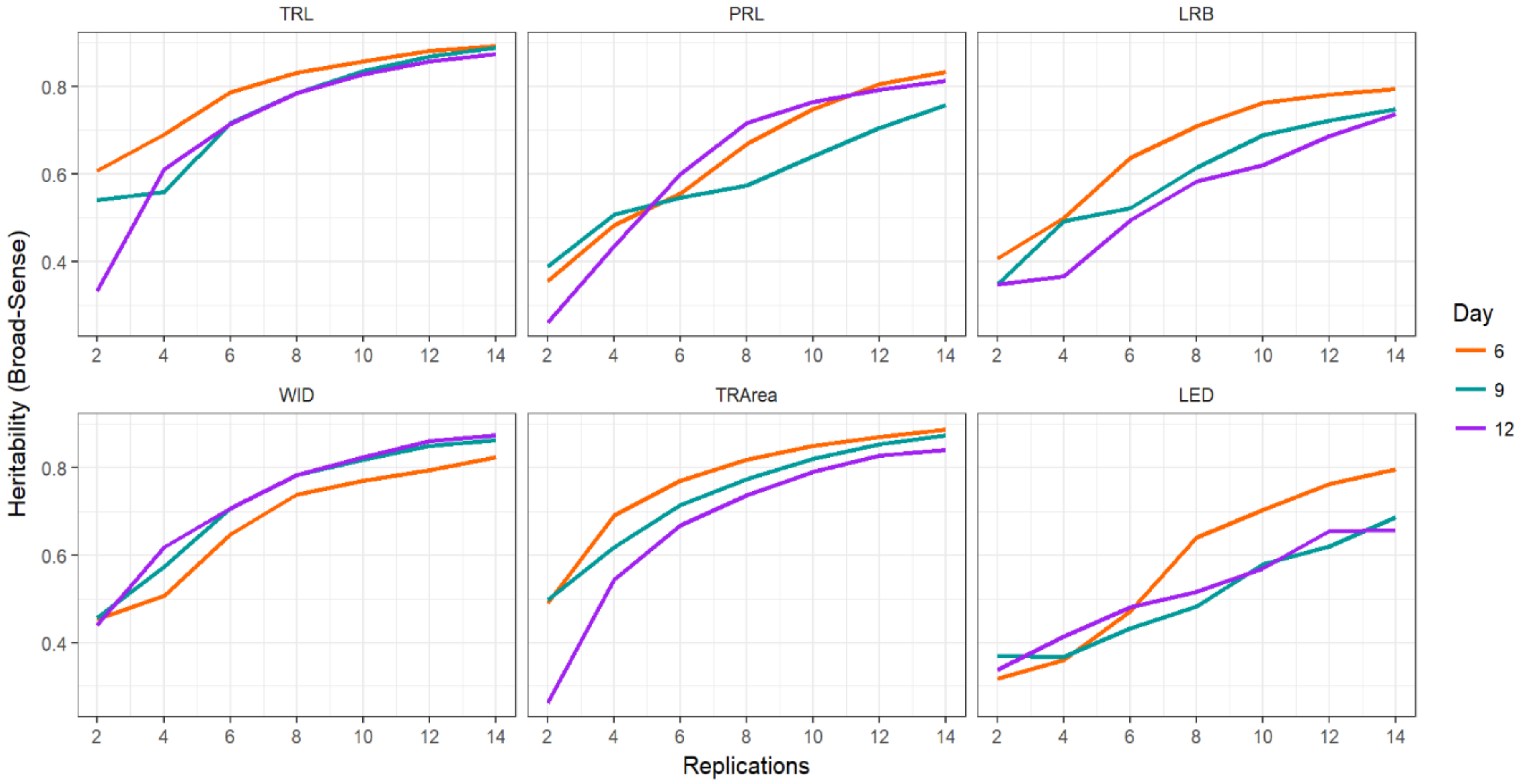

Supplementary Figure 3

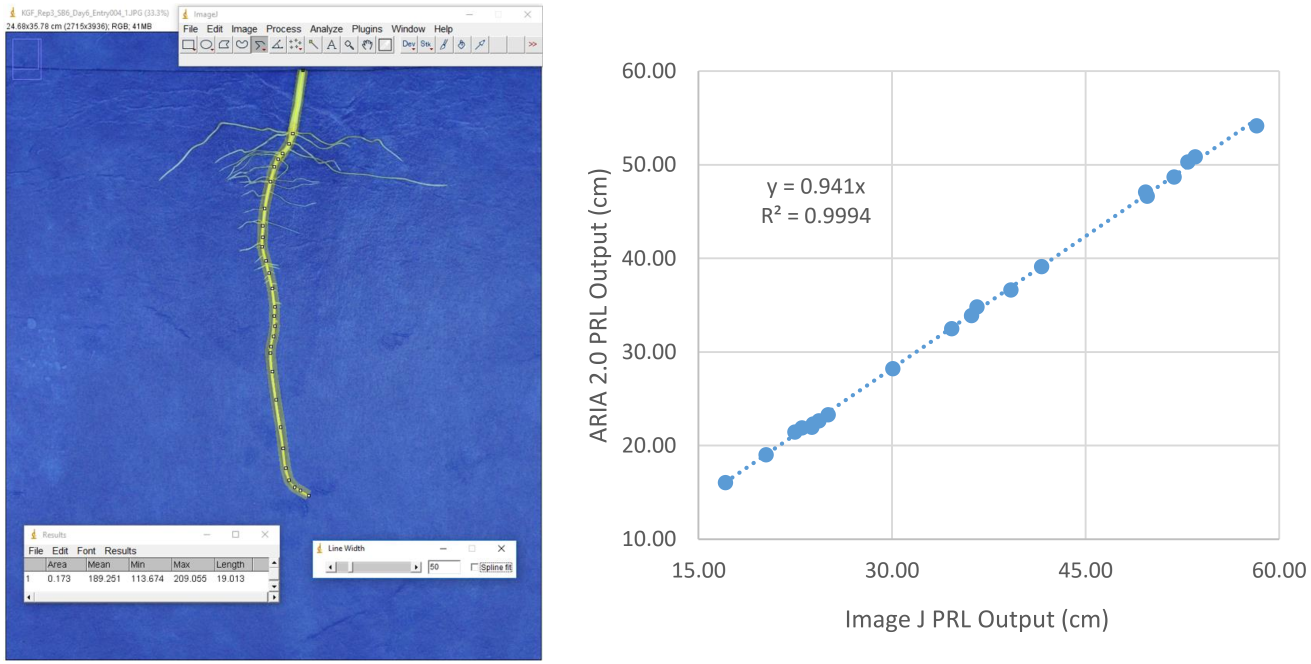

Supplementary Figure 4

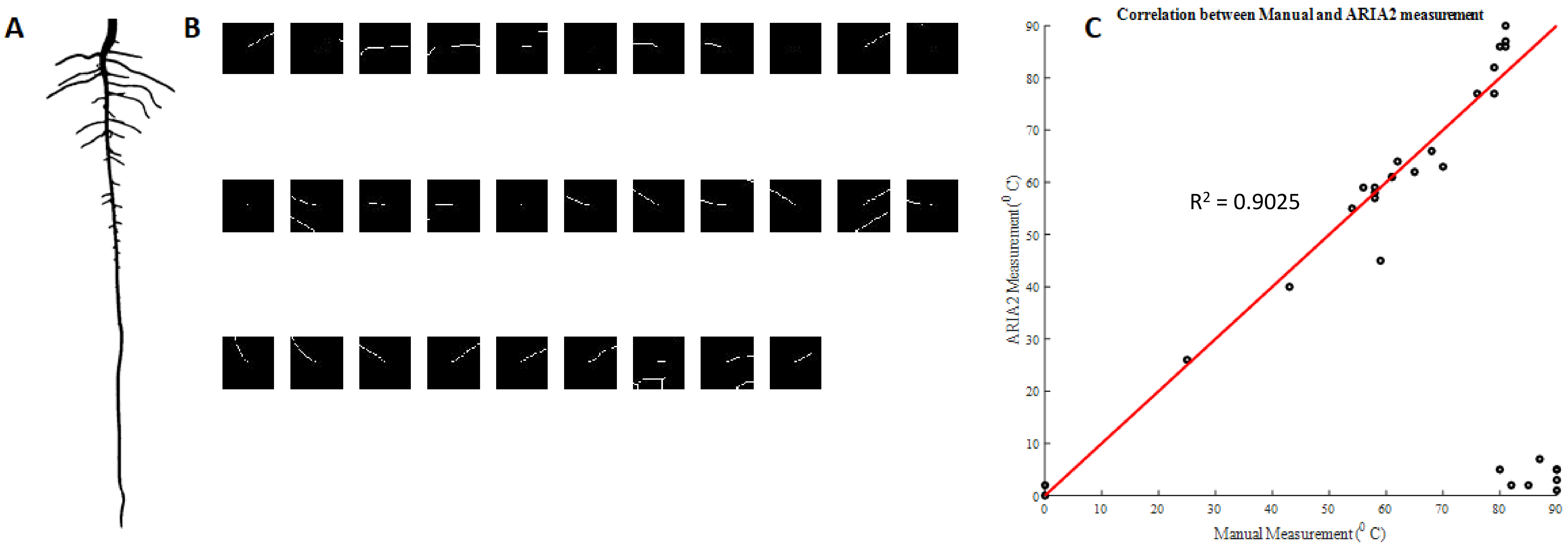

Supplementary Figure 5a, b, c

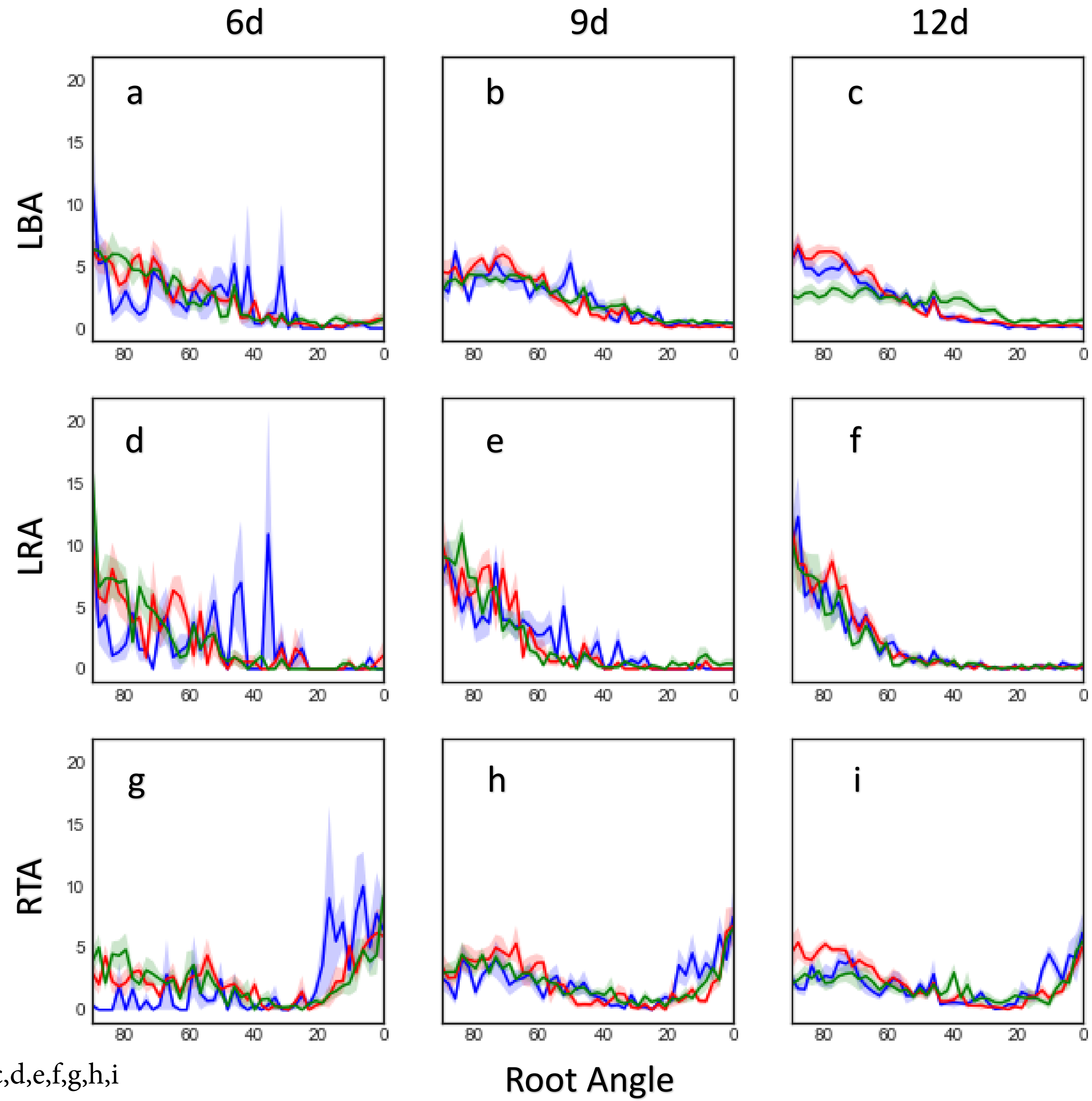

Supplementary Figure 6 a,b,c,d,e,f,g,h,i

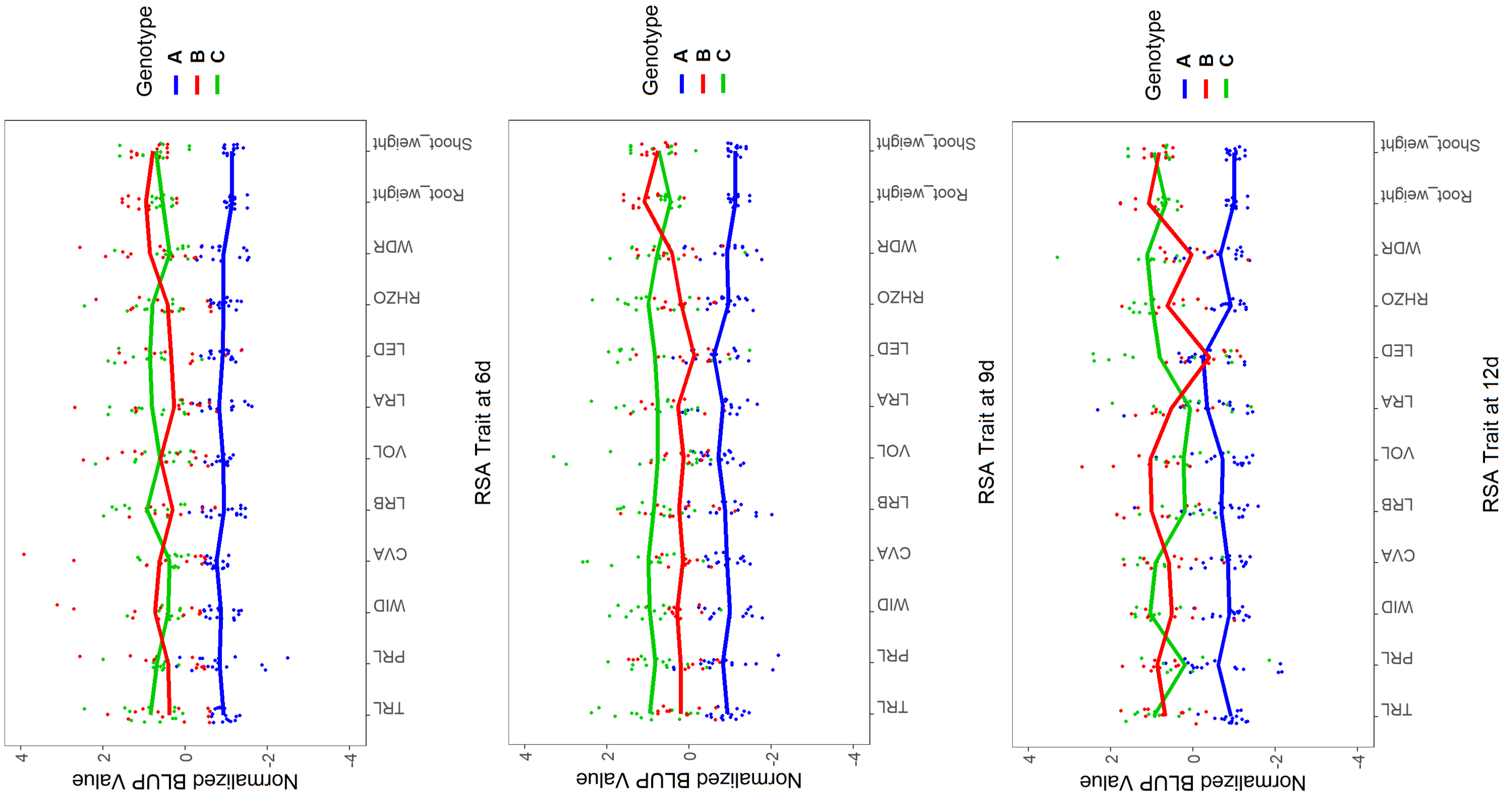

Supplementary Figure 7

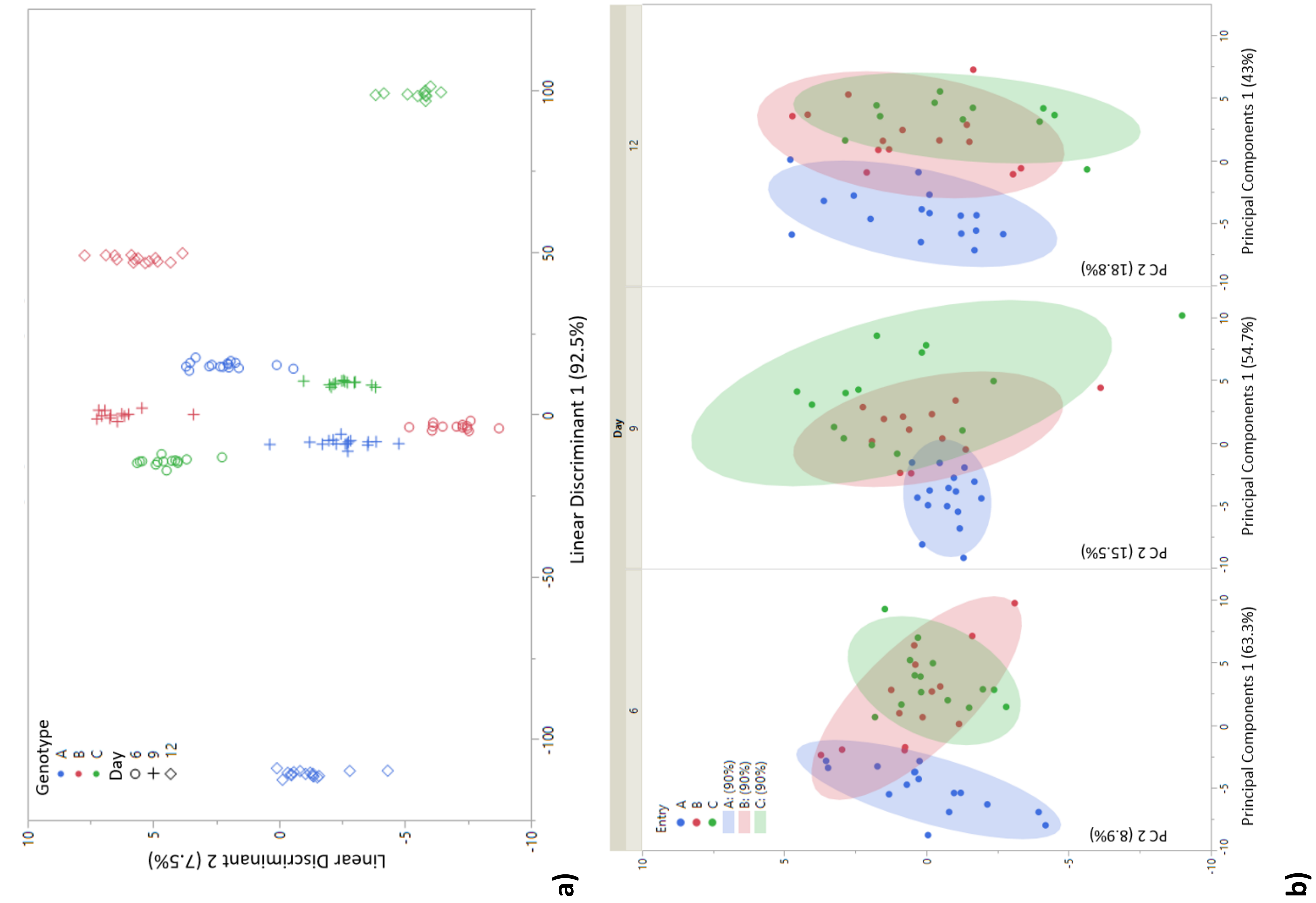

Supplementary Figure 8a,b
